# Genome-wide association analyses of quantitative disease resistance in diverse sets of soybean [*Glycine max* (L.) Merr.] plant introductions

**DOI:** 10.1371/journal.pone.0227710

**Published:** 2020-03-20

**Authors:** William Rolling, Rhiannon Lake, Anne E. Dorrance, Leah K. McHale

**Affiliations:** 1 Center for Applied Plant Science and Center for Soybean Research, The Ohio State University, Columbus, Ohio, United States of America; 2 Department of Horticulture and Crop Science, The Ohio State University, Columbus, Ohio, United States of America; 3 Department of Plant Pathology, The Ohio State University, Wooster, Ohio, United States of America; University of Guelph, CANADA

## Abstract

*Phytophthora sojae* is one of the costliest soybean pathogens in the US. Quantitative disease resistance (QDR) is a vital part of Phytophthora disease management. In this study, QDR was measured in 478 and 495 plant introductions (PIs) towards *P*. *sojae* isolates OH.121 and C2.S1, respectively, in genome-wide association (GWA) analyses to identify genetic markers linked to QDR loci (QDRL). Populations were generated by sampling PIs from the US, the Republic of Korea, and the full collection of PIs maintained by the USDA. Additionally, a meta-analysis of QDRL reported from bi-parental studies was done to compare past and present findings. Twenty-four significant marker-trait associations were identified from the 478 PIs phenotyped with OH.121, and an additional 24 marker-trait associations were identified from the 495 PIs phenotyped with C2.S1. In total, 48 significant markers were distributed across 16 chromosomes and based on linkage analysis, represent a total of 44 QDRL. The majority of QDRL were identified with only one of the two isolates, and only a region on chromosome 13 was consistently identified. Regions on chromosomes 3, 13, and 17 were identified in previous GWA-analyses and were re-identified in this study. Five QDRL co-localized with *P*. *sojae* meta-QDRL identified from QDRL reported in previous biparental mapping studies. The remaining regions represent novel QDRL, in the soybean-*P*. *sojae* pathosystem and were primarily identified in germplasm from the Republic of Korea. Overall, the number of loci identified in this study highlights the complexity of QDR to *P*. *sojae*.

## Introduction

Phytophthora root and stem rot [1] is an economically significant disease of soybean [*Glycine max* (L.) Merr]. Infection occurs under favorable environmental conditions for disease development, including saturated soils and temperatures around 25 °C [2–4]. Under these conditions, zoospores of *P*. *sojae* chemotactically swim towards soybean roots [5,6]. Successful infection of susceptible plants results in seed rot and damping off at early growth stages, and wilting, stem lesions, and plant death at later growth stages. Genetic resistance is considered the most effective strategy for preventing or reducing the impact of Phytophthora diseases [4,7].

Soybean-breeding programs have relied on resistance conferred by single, dominantly inherited *Resistance to Phytophthora sojae* (*Rps*)-genes. More than 30 *Rps*-genes/alleles have been mapped (S1 Table) [8–25]. The effectiveness of *Rps*-mediated resistance is limited as these genes only confer immunity towards specific races of *P*. *sojae*. Over time this results in selection within pathogen populations, leading to adaptation [3,4] and limited *Rps*-gene lifespans of 8–20 years [26]. A recent survey completed in the North Central region of the US demonstrated that none of the deployed *Rps*-genes confer resistance to all *P*. *sojae* isolates, with field populations having complex virulence patterns [27].

Quantitative disease resistance (QDR) is an additional form of genetic resistance, functioning through the action of multiple genes each conferring a portion of the overall phenotype [28–30]. Though QDR does not confer complete immunity, the lack of race-specificity is predicted to place less selection on pathogen populations; thus, QDR is expected to be more durable than race-specific resistance [31–33]. For example, wheat growers have used QDR for stripe and leaf rust resistance since the early 20th century, with no observed increase in pathogen virulence [34]. Similarly, QDR for the rice pathogen *Magnaporthe oryzae* contributed by *Pi21* has conferred effective resistance for over a century [35]. Used in combination, QDR can also increase the durability of *Resistance-*gene (*R*-gene) mediated resistance as highlighted by examples of pepper-potato virus Y [36] and root-knot nematode pathosystems of potato [3,37].

In the soybean-*P*. *sojae* pathosystem, QDR has also played an important role in disease resistance breeding [3]. QDR protects soybean yields when *Rps-*gene(s) are ineffective and the environment is conducive to disease [7,38]. Breeding efforts to introduce QDR (and other traits) for cultivar development involve large populations. Field testing can be misleading as the pathotypes of *P*. *sojae* populations within fields are highly variable, the presence of an *Rps*-gene could mask the effects of QDR [39], and QDR is not expressed within the first five days following germination [40]. Marker-assisted selection (MAS) can provide an alternative method for selecting cultivars with higher levels of QDR. Thus, studies to map the genetic positions of QDR can be applied to increase the efficiency of selection for these traits via MAS [41].

Numerous biparental mapping studies identified QDR loci (QDRL) towards *P*. *sojae* in US cultivars Conrad, Sloan, and V71-370 [42–48]. Recently, the soybean nested associated mapping populations were used to study *P*. *sojae* resistance, identifying four QDRL [49]. In addition to US cultivars, QDRL have been mapped in landraces originating from China and the Republic of Korea (South Korea, SK) using biparental mapping populations derived from crosses with eight accessions [50–54]. The majority of these mapping studies identify several QDRL within each population; most were small-effect loci explaining less than 10% of the variation (S2 Table).

Mapping studies have also been completed using genome-wide association (GWA) analyses. This includes a study of 797 plant introductions (PIs) from SK [55] which had previously been shown to be a source of *Rps*-genes and high levels of QDR against *P*. *sojae* [56], 175 Chinese soybean breeding lines [57], and 279 breeding lines from the Yangtze-Huai Chinese breeding program [58]. Recently, a collection of 169 Brazilian cultivars was used to identify QDRL in regional germplasm [59]. Similar to loci identified by biparental mapping, these GWA studies identified small-effect QDRL.

Although soybean growers deploy QDR to *P*. *sojae* in the US [3], US germplasm has never been evaluated in a GWA analysis for QDR to *P*. *sojae*. A population derived from US germplasm could be useful in identifying alleles in germplasm most adapted for the region. Though alleles from adapted germplasm may be easier to introgress or maintain in elite cultivars, identifying novel QDRL from diverse germplasm remains a crucial aspect of resistance breeding. Thus, the objective of the current research was to complete genome-wide association (GWA) analyses by sampling US, SK, and the full diversity of the USDA soybean PI collection. Two isolates were used to assay QDR because *P*. *sojae* is diverse [27] and previous studies have mapped different QDRL depending on the isolate [47,48].

We phenotyped 547 (495 used in GWA analysis) PIs for QDR using isolate C2.S1 of *P*. *sojae* and also measured QDR in a different set of 549 PIs (478 used in GWA analysis) with the second isolate OH.12108.6.3 (OH.121) of *P*. *sojae*. In total, 48 SNPs associated with QDR to *P*. *sojae* were identified and consolidated into 44 QDRL, with 21 QDRL identified with OH.121 and 23 QDRL identified with C2.S1. In addition to GWA analyses, we completed a meta-QDRL analysis to consolidate previous results and identify any colocalization between the loci identified in this study by GWA analysis and those QDR previously identified from biparental mapping studies. The 44 QDRL reported here represent a mixture of both novel QDRL and those that confirm results of previous mapping studies of this complex pathosystem.

## Materials and methods

### Genotypic data

We used publically available genotypic data from the SoySNP50K iSelect BeadChip [60] downloaded from Soybase [61] (www.soybase.org/snps). In total, 42,509 SNP data were available for 20,087 PIs. Monomorphic markers, markers with > 5% missing data, and markers with a minor allele frequency of < 5% were removed prior to analyses. For the remaining SNPs, missing marker data was imputed with fastPHASE [62]. Using the 1096 PIs phenotyped in this study, genome-wide linkage disequilibrium (LD) decay was estimated for both euchromatic and heterochromatic regions by plotting physical distance vs. linkage (r^2^).

### Germplasm diversity and selection

FastStructure [63] population analysis using default settings was used to identify related populations. PIs were assigned to populations using a membership coefficient threshold (Q-value) of ≥ 0.7. We sampled the USDA collection to create two distinct sets of PIs, phenotyped with either *P*. *sojae* isolate OH.121 (OH set) or C2.S1 (C2 set). PIs were represented once and were phenotyped with only C2.S1 or OH.121, not both isolates of *P*. *sojae* because a limited number of seeds were provided by the germplasm center, limiting the assays to one isolate per PI. Three populations were represented within the C2 set and OH set. The first populations were randomly sampled twice from the 20,087 PIs to create the C2-GRIN and OH-GRIN populations. Population structure analyses identified a population of 817 PIs primarily consisting of US-North-Central region PIs and 1805 PIs mainly originating from SK. The PIs originating from the US were randomly sampled twice to create the C2-US and OH-US populations. Similarly, the SK originating PIs were randomly sampled twice to create the C2-SK and OH-SK populations.

For the C2 set of PIs (consisting of the C2-US, C2-SK, and C2-GRIN populations), 547 PIs were phenotyped, and 495 PIs used for GWA analysis (S3 Table). The three populations had samplings of 180 PIs for the US population, 162 PIs for the SK population, and 153 PIs sampled from the GRIN population. The OH set of PIs (consisting of the OH-US, OH-SK, and OH-GRIN populations) had 549 PIs, which were unique from those included in the C2 set. The OH set included 478 PIs (S4 Table) in GWA analysis composed of 170 US PIs, 158 SK PIs, and 150 PIs sampled from the GRIN population.

In addition to the PIs, cultivars with characterized QDR against *P*. *sojae* (‘L83-570—*Rps3a*’, ‘Williams 79—*Rps1c*’, ‘Conrad—*rps*’, ‘Sloan—*rps*’, ‘Resnik—*Rps1k*’,‘OX20-8—*Rps1a*’), were included as checks. The *P*. *sojae* isolates were expected to be virulent on these lines despite the presence of *Rps-*genes, with the exception of OH.121 on ‘Williams 79—*Rps1c*’. Before disease assays, all seeds were surface sterilized using chlorine gas [64].

### Quantitative disease resistance assays

Phenotyping of QDR was completed using the layer test [65] between February and April 2018 with 16 hrs of supplemental lighting and temperatures maintained between 18°C and 26°C. Briefly, a 950mL Styrofoam cup was filled from bottom to top with: (1) 2.5 cm layer of coarse vermiculite, (2) 7.6 cm of fine vermiculite, (3) a two-week-old culture of *P*. *sojae* grown on dilute lima bean agar in a 100 mm x 15 mm Petri dish or for non-inoculated treatments 0.6 cm of fine vermiculite, (4) 3.8 cm of fine vermiculite, (5) 8 seeds, and (6) a final layer of coarse vermiculite to cover the seeds. Each block of the experiment was maintained over a three-week period during which the cups were watered twice a day.

The data collected for GWA analyses included an Inoculated Root Rot Score (IRRS), Inoculated Root Weight (IRW), Inoculated Shoot Weight (ISW), Inoculated Plant Height (IPH), Non-inoculated Root Weight, Shoot Weight and Plant Height (NRW, NSW, NPH). The plant heights (IPH, NPH) were collected from three representative plants and averaged. The IRRS score was scored on a scale of 1–9 [65]. Change in root and shoot weight (ΔRW, ΔSW) were calculated by subtracting non-inoculated weights from inoculated weights for each PI line.

We used an incomplete block design with PIs randomized with restriction so that each block contained an inoculated and non-inoculated experimental unit (Styrofoam cup) for each PI represented in a block. Each block consisted of ~183 PIs, with 8 seeds per experimental unit. Check cultivars were included in all blocks. Two replicates were completed for each PI resulting in six blocks for both the C2 set and OH set, or 12 blocks total.

Best Linear Unbiased Predictor (BLUP) values and Best Linear Unbiased Estimators (BLUEs) were calculated for PIs and checks, respectively, for each trait measured in the experiment using the R package [66] lme4 [67]. The equation used for each trait was *Y*_*hijk*_ = *μ* + *C*_*h*_ + *L*(*C*)_*hi*_ + *B*(*R*)_*jk*_ + *ε*_*hijk*_, where *μ* is the grand mean, *C*_*h*_ is the effect *h*th class representing one of the six checks or an experimental PI, *L*(*C*)_*hi*_ is the genotypic effect of each line, *B*(*R*)_*jk*_ is the *j*th block effect within *k*th rep, and *ε* represents the residual. Broad-sense heritability (H^2^) was calculated for each trait *H*^2^ = *σ*_*g*_^2^/(*σ*_*g*_^2^ + *σ*_*ε*_^2^/*r*), where *σ*_*g*_^2^ (genetic variance), *σ*_*ε*_^2^ (error variance), and r (number of replicates).

### Phytophthora isolates and *Rps-*gene assays

The focus of this study was on QDR, therefore isolates with complex virulence pathotypes were used to limit *Rps*-gene responses masking QDR. The Ohio isolates OH.12108.6.3 (OH.121) (*vir 1a*, *1b*, *1d*, *1k*, *2*, *3a*, *3c*, *4*, *5*, *6*, *7*, *8*) and C2.S1 (*vir 1a*, *1b*, *1c*, *1d*, *1k*, *2*, *3a*, *3c*, *4*, *5*, *6*, *7*, *8*) were used in the QDR assays. The virulence of these isolates had previously been assessed [55], but to ensure the pathotypes were consistent after storage, virulence was reassessed here.

To ensure that we were evaluating QDR and not *Rps*-mediated resistance, we evaluated the 547 lines in the C2 set and the 549 lines from the OH set with the hypocotyl test. Due to limited seed number, eight seedlings were grown and two replicates completed. Scoring thresholds of 70% and 30% were applied. When no lesion developed on 70% of the plants, it was considered a *Rps*-gene response and removed from GWA analyses. PIs that developed a lesion 70–90% of the time were further scrutinized by checking the scores in the layer test to ensure a lack of an *Rps*-gene response, and PIs with IRRS scores less than 1.5 were removed from the GWA analyses.

### GWA analyses

The C2 set and the OH set were each analyzed separately; four GWA analyses were completed for C2 and OH set, for a total of 8. An analysis was completed using all PIs for a set as well as the three populations (C2-US, C2-SK, C2-GRIN, OH-US, OH-SK, and OH-GRIN) that comprise each set. GWA analyses were implemented using the R [66] package GAPIT [68] using a multiple locus mixed model (MLMM). General linear model, mixed linear model, compressed mixed linear model, and FarmCPU were also tested, but did not yield appropriate Q-Q plots or were not as able to resolve significant associations (S1 Fig). When implemented in GAPIT, MLMM does not readily provide an estimate of percent variation explained. Instead, PVE was estimated using CMLM. The Q-matrix resulting from fastStructure analyses was used as a covariate. The significance threshold was determined using a modified Bonferroni adjustment by calculating the M_eff_ using SimpleM [69]. Genome-wide significance thresholds were determined by α/M_eff_ where α = 0.05.

Haploview [70] was used for haplotype block assembly using the four-gamete rule [71]. A haplotype block possessing a marker(s) with a significant marker-trait association was considered a single QDRL. Haplotype blocks for markers that were unlinked to other markers were delimited according to the positions of the flanking markers.

### Meta-QDRL analysis

We collected pertinent information for reported QDRL towards *P*. *sojae* identified prior to Jan 1, 2019. Collected data included (1) linkage maps, (2) R^2^ of genetic effect, (3) significance (LOD), (4) linkage group, (5) confidence interval (CI), (6) predicted position of QDRL, (7) generation and (8) size of populations from 16 biparental mapping studies (S2 Table). Due to inconsistent reporting of these values, estimates were made for LOD values and confidence intervals (CIs) when not provided. LOD values were calculated using the formula LOD = R^2^/1.5. If the CI was not provided it was calculated using the equation CI = 163/(N x R^2^) where N is the population size [72].

Meta-analysis was completed using the software BioMercator V3.1 [73,74]. The initial step was to create a project-specific consensus map from genetic maps found in the biparental studies; this was aided by the inclusion of the consensus map [75] downloaded from Soybase [61]. QDRL were projected onto the project-specific consensus map. Studies prior to 2010 were removed and only one mapping study per generation of each biparental cross was included. The projected QDRL were used to identify position and CI based on Akaike-type criteria values. The physical positions of meta-QDRL were determined by selecting the markers flanking the meta-QDRL and locating the positions of these markers in the Wms.82 genome (Wms.82.a2.v1) [76].

## Results

### Population structure analysis and linkage disequilibrium

**Fig 1:**
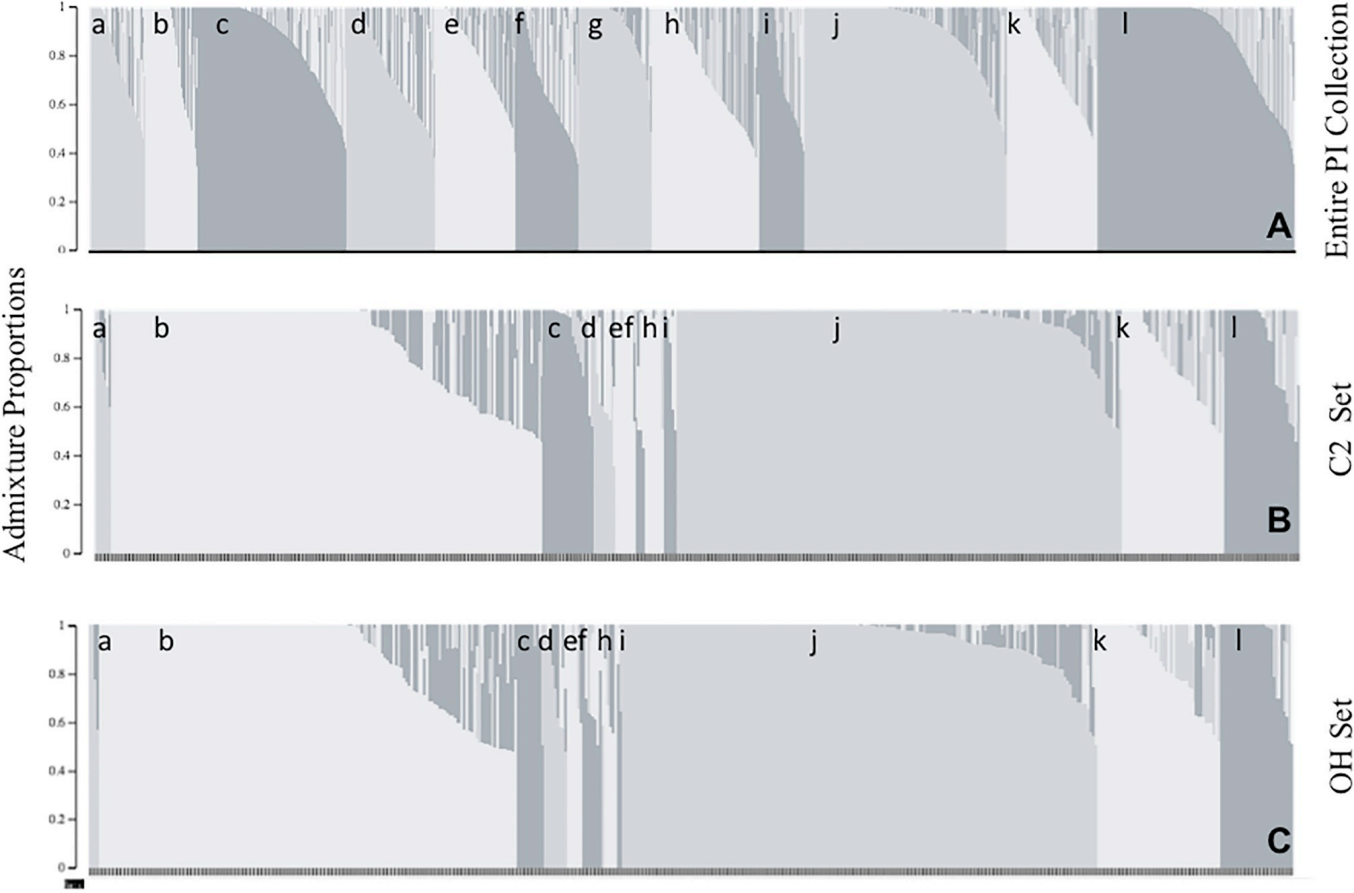
Estimated population structure of soybean plant introductions (PIs) genotyped with the SoySNP50K iSelect BeadChip. (A) Population structure analysis of the 20,087 genotyped PIs maintained by the USDA Soybean Germplasm Center (Urbana, IL) was carried out at *k* = 12. (B) The C2 set consists of 495 PIs phenotyped with *P*. *sojae* isolate C2.S1. (C) The OH set consists of 478 PIs phenotyped with *P*. *sojae* isolate OH.121. The 12 population-groups identified by fastStructure are indicated by lower case letters. The United States (US) and South Korean (SK) population-groups of interest are indicated with labels “b” and “j”, respectively. To create C2-US, C2-SK, OH-US, and OH-SK, the US and SK populations-groups (b, j) were sampled.

The population structure of the USDA germplasm collection was analyzed in order to generate population-groups from which our GWA populations would be sampled. Population structure analyses of the 20,087 PIs with SoySNP50K genotypic data resulted in marginal likelihood estimations increasing with model complexities (number of population-groups) from 1 to 20 (S5 Table). A local maximum was present at *k* = 12, describing twelve major population-groups of soybean PIs (Fig 1A).

Two population-groups were identified that primarily consisted of PIs originating from the North Central US and SK, these were specifically sampled. PIs originating from SK have previously been shown to be a rich source of QDR alleles [55,56], providing novel QDR alleles to breeders that can greatly benefit resistance breeding [30]. The US population-group is a potential source of QDR alleles in genetic backgrounds adapted to the North Central regions of the US or alleles already deployed in US cultivars. To generate the six populations for our GWA analyses, we sampled the SK population-group to create the C2-SK and OH-SK populations and sampled the US population-group to create the C2-US and OH-US populations. Finally, the full USDA germplasm collection was sampled to generate the C2-GRIN and OH-GRIN populations to access a broader diversity, potentially providing novel, under-utilized QDR alleles.

The combination of the C2-SK, C2-US, and C2-GRIN populations were referred to as the C2 set. Likewise, the OH set was comprised of the OH-SK, OH-US, and OH-GRIN populations. Eleven of the 12 population-groups identified by the fastStructure analysis of the 20,087 PIs in the germplasm collection were represented in both the C2 set and OH set (Fig 1B and 1C). Additional population structure analyses were completed to identify subpopulations in the PIs comprising C2-US and OH-US as well as the C2-SK and OH-SK populations. These analyses identified six and nine subpopulations within the US and SK populations, respectively (S2 and S3 Figs). The majority of the subpopulations are present in the respective C2-US and OH-US populations and C2-SK and OH-SK populations, with only slightly different proportions of each subpopulation represented (S2 and S3 Figs).

A total of 33,234 (C2 set) and 34,248 (OH set) SNP-markers were used in GWA analyses. There was an average density of one marker every 29 kb for both the C2 and OH sets. Due to linkage disequilibrium (LD), SNP-markers could be consolidated to an effective marker number [69] of approximately 11,000 in the OH set and C2 set, as well as the OH-GRIN and C2-GRIN populations. The effective marker numbers were nearly 5,000 for the C2-SK and OH-SK populations, 4,051 in C2-US population, and 3,291 in the OH-US population (S4 Fig). In the combined 974 individuals from the C2 and OH sets, where LD is expected to be lower than the less diverse populations (i.e., C2-US, C2-SK, OH-US, and OH-SK) [77], LD decayed to an r^2^ of 0.2, a common threshold of significant LD [78], at an approximate distance of 350kb in euchromatic regions and greater than 500kb in heterochromatic regions (S4 Fig). Given that each population has an estimated marker density < 300 kb/marker, we anticipate that the majority of the soybean genome was captured in each GWA analysis (S4 Fig).

### Quantitative disease resistance of PIs in the C2 set

A total of 547 PIs were phenotyped with isolate C2.S1, of which 52 were subsequently removed from analyses due to an *Rps-*gene response or missing data. For the remaining 495 PIs that comprised the C2 set, best linear unbiased predictor (BLUP) values were calculated from the raw measurements of inoculated and non-inoculated traits. The BLUP values for NRW, NSW, and NPH had normal distributions, while IRRS, IRW, and ISW had nearly normal distributions with a slight positive skew and ΔRW, ΔSW and NPH had nearly normal distributions with a slight negative skew (S5 Fig). For the C2 set, all of the inoculated traits had significant genetic variance and moderate to moderately high broad-sense heritability, ranging from 0.52 for ΔRW to 0.78 in IRW (Table 1).

**Table 1:** Heritability and genetic variance for layer test traits with *Phytophthora sojae* isolates C.2.S.1 and OH.121 for the C2 and OH sets of plant introductions, respectively.

	C2 set		OH set	
Trait^a^	*H*^2^^b^	σ_g_^2^^c^	*H*^2^	σ_g_^2^
IRRS	0.58	0.94***^d^	0.65	1.98*
IRW	0.78	0.19***	0.45	0.10*
ISW	0.65	0.23***	0.68	0.27***
IPH	0.58	7.70***	0.77	14.00***
ΔRW	0.52	0.11***	0.38	0.09***
ΔSW	0.56	0.12***	0.43	0.16***
NRW	0.75	0.23***	0.39	0.14*
NSW	0.66	0.25***	0.65	0.30***
NPH	0.55	6.57***	0.63	8.12***

^a^, IRRS, inoculated root rot score; IRW, inoculated root weight; ISW, inoculated shoot weight; IPH, inoculated plant height; ΔRW, change in root weight; ΔSW, change in shoot weight; NRW, non-inoculated root weight; NSW, non-inoculated shoot weight; NPH, non-inoculated plant height

^b^, *H*^2^, broad-sense heritability

^c^, σg^2^, genetic variance;

^d^, *, significant at P value < 0.05;

***, significant at P value < 0.001.

Within the C2 set, the C2-US, C2-SK, and C2-GRIN populations had comparable average BLUP scores for each trait (S6 Fig). Despite similar means, subtle trends in the distributions of BLUP values were present; the US population had significantly higher BLUP values for NRW and NSW, indicating that these PIs were larger (S6 Fig). Consistent with previous studies, the C2-SK population had the lowest BLUP values for IRRS (most resistant) and highest BLUP values for IRW, ISW, and IPH of the three populations, once again demonstrating the high resistance levels found in SK germplasm. The SK and GRIN populations had greater genetic variance and a larger standard deviation around the means for all traits (S6 Fig). The C2-US population had the lowest average heritability (*H*^*2*^ = 0.39) and genetic variance, though still significant for all traits except ΔRW and ΔSW. Both the C2-SK and C2-GRIN populations also had significant genetic variance for all traits and higher average broad-sense heritability between 0.73 and 0.66, respectively (S6 Table).

In the C2 set, there was significant correlation between nearly all traits (Table 2). Correlations were strongest among traits of the same type (inoculated, non-inoculated, or combined). Correlations among inoculated traits were moderate to high, ranging from -0.53 for IRRS (lower score is more resistant) and IPH to 0.87 for ISW and IRW. The correlation between the combined traits ΔSW and ΔRW was also high at 0.79, and correlations among non-inoculated traits were moderate to high, ranging from 0.59 for NPH and NRW to 0.83 for NSW and NRW. For the most part, correlations between different types of traits were low to moderate, ranging from -0.21 for ΔRW and NPH to 0.56 for ISW and NSW. However, IRRS was not significantly correlated to the non-inoculated traits, indicating that IRRS is independent of non-inoculated plant size and weights.

**Table 2:** Significant correlations between layer test traits assessed within the C2 set (unshaded) and OH sets (shaded) of plant introductions.

	Inoculated				Combined		Non-inoculated		
**Trait**^a^	**IRRS**	**IRW**	**ISW**	**IPH**	**ΔRW**	**ΔSW**	**NRW**	**NSW**	**NPH**
**IRRS**		0.63	-0.54	-0.53	-0.57	-0.59	--^b^	--	--
**IRW**	-0.71		0.87	0.73	0.38	0.37	0.48	0.51	0.38
**ISW**	-0.65	0.83		0.75	0.30	0.43	0.45	0.56	0.36
**IPH**	-0.77	0.76	0.72		0.23	0.31	0.37	0.41	0.43
Δ**RW**	-0.38	0.38	0.26	0.25		0.79	-0.43	-0.31	-0.21
Δ**SW**	-0.51	0.44	0.48	0.39	0.58		-0.27	-0.35	-0.24
**NRW**	--	0.41	0.36	0.36	-0.45	-0.43		0.83	0.59
**NSW**	--	0.40	0.51	0.34	-0.30	-0.49	0.78		0.69
**NPH**	--	0.33	0.3	0.43	-0.19	-0.35	0.63	0.65	

All displayed correlations were significant at a P value of < 0.001

^a^, IRRS, inoculated root rot score; IRW, inoculated root weight; ISW, inoculated shoot weight; IPH, inoculated plant height; ΔRW, change in root weight; ΔSW, change in shoot weight; NRW, non-inoculated root weight; NSW, non-inoculated shoot weight; NPH, non-inoculated plant height

^b^, no significant correlation.

### Quantitative disease resistance of PIs in the OH set

A total of 549 PIs were phenotyped with isolate OH.121, and 71 PIs were removed from subsequent analyses due an *Rps-gene* response or missing data. Thus, the final OH set consisted of 478 PIs. The BLUP values for NRW and NSW had normal distributions (S7 Fig). The other traits had nearly normal distributions with a slight positive skew to IRRS, IRW, and ISW; a slight negative skew for IPH and NPH; and heavy tailing in the ΔRW and ΔSW traits (S7 Fig). All nine traits had significant genetic variance (Table 1) with moderate to high heritability, ranging from 0.38 for ΔRW to 0.77 for IPH. Both the heritability and genetic variance were comparable between isolates, with OH.121 traits on average having slightly higher genetic variance, higher residual variance, and marginally lower heritability (Table 1).

The average BLUP values of the three populations comprising the OH set (OH-GRIN, OH-SK, OH-US) were very similar for each trait, with a greater standard deviation of BLUP values in the OH-GRIN and OH-SK populations relative to the OH-US population (S7 Fig). Genetic variance was lowest in the US population, and heritability was highest in the SK population. The US and SK populations had similar BLUP values for IRRS, IRW, ISW, and IPH and were significantly more resistant than the GRIN population. The US population had significantly higher BLUP values for the non-inoculated traits (sans NSW compared to SK) indicating, once again, that the US population averaged larger plants than the SK and GRIN populations.

There were significant correlations among nearly all traits in the OH set (Table 2). Correlations were strongest among traits of the same type (inoculated, non-inoculated, or combined). Correlations among inoculated traits were moderate to high, ranging from -0.65 for IRRS and ISW to 0.83 for ISW and IRW. The correlation between the combined traits ΔSW and ΔRW was moderate at 0.58, and correlations among non-inoculated traits were moderate to high, ranging from 0.63 for NPH and NRW to 0.78 for NSW and NRW. For the most part, correlations between different types of traits were low to moderate, ranging from -0.19 for ΔRW and NPH to -0.51 for ISW and NPH. However, similar to data from the C2 set, IRRS was not significantly correlated to the non-inoculated traits, indicating that IRRS is independent of non-inoculated plant size and weights.

### Genome-wide association analyses

**Fig 2:**
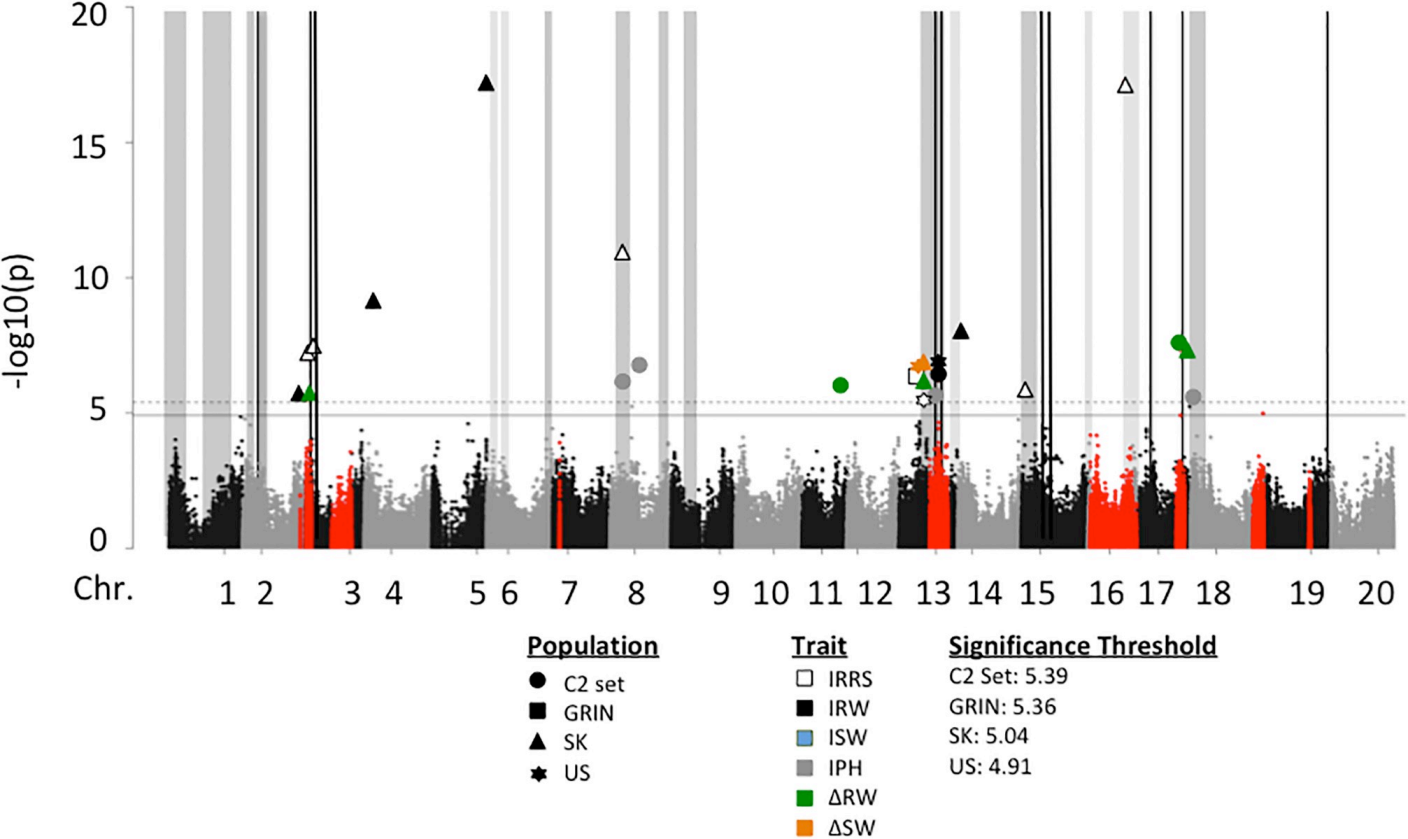
Manhattan plot of genome wide association of layer test traits from the C2 set. Marker associations for the full C2 set and the C2-GRIN, C2-SK, and C2-US populations for each trait are overlaid. For significant associations, markers for each population and trait are differentiated by the shape and color of the marker, respectively. Significance thresholds were calculated for each population using SimpleM, only the highest (C2 set) and lowest (C2-US) thresholds are displayed. Red shading of non-significant markers indicates the genetic regions associated with known *Rps*-genes. Genomic positions previously identified significantly associated with quantitative resistance to *P*. *sojae* in previous GWA analyses are indicated by the black vertical bars. Grey highlighted regions represent meta-QDRL, with the darker grey highlight indicating QDRL identified in exotic germplasm, and the lighter grey representing QDRL identified from US cultivars.

**Fig 3:**
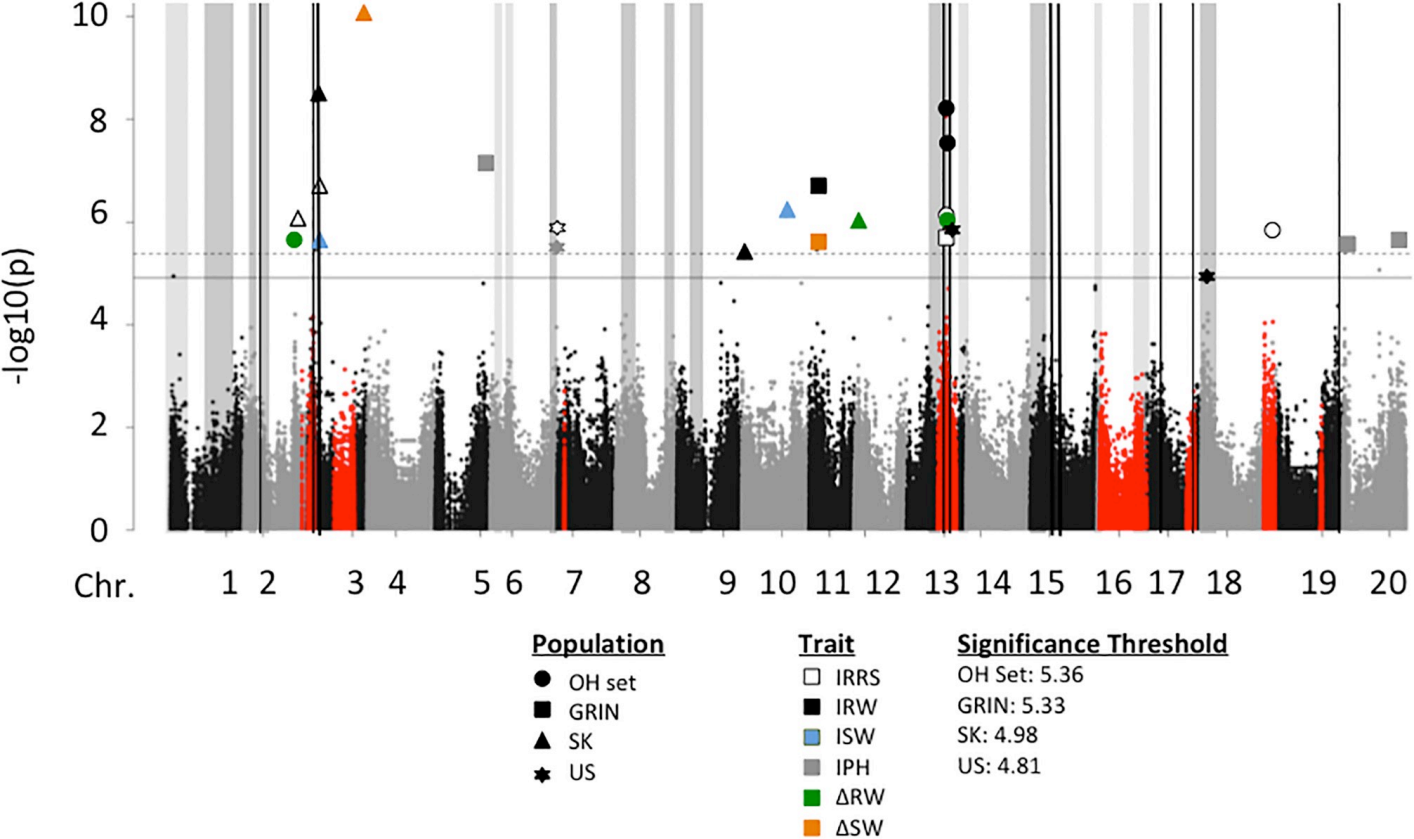
Manhattan plot of genome wide association of layer test traits from the OH set. Marker associations for the full OH set and the OH-GRIN, OH-SK, and OH-US populations for each trait are overlaid. For significant associations, markers for each population and trait are differentiated by the shape and color of the marker, respectively. Significance thresholds were calculated for each population using SimpleM, only the highest (OH set) and lowest (OH-US) thresholds are displayed. Genomic locations of *Rps*-genes, regions identified as significantly associated with quantitative resistance to *P*. *sojae* in previous GWA analyses, and meta-QDRL are indicated as described for Fig 3.

A total of 48 significant marker-traits associations were identified on 16 chromosomes (Tables 3 and 4; Figs 2 and 3). Markers in LD with significant markers were considered the region most likely to contain the allele contributing to QDR and were used to delimit QDRL. Some markers were associated with more than one trait or were within the same haplotype block; therefore, the 48 marker-trait associations were consolidated into 44 QDRL.

**Table 3:** Quantitative disease resistance loci (QDRL) mapped in the C2 set.

Name^a^	Pop^b^	Trait^c^	-log_10_(p)	PVE^d^	Marker^e^	Flanking positions^f^
C2-02-1	C2-SK	IRW	5.49	2.78	ss715582994	43367206–43440684
C2-03-1	C2-SK	IRRS	7.02	6.88	ss715586961	786873–821859
C2-03-2	C2-SK	ΔRW	5.48	7.03	ss715586992	821859–875151
C2-03-3	C2-SK	IRRS	7.43	4.04	ss715585633	3691222–3895958
C2-04-1	C2-SK	IRW	9.03	5.52	ss715589155	6514173–6682383
C2-05-1	C2-SK	IRW	17.32	5.71	ss715591632	41780982–42090709
C2-08-1	C2 set	IPH	6.1	2.79	ss715602597	5472166–5709053
C2-08-2	C2-SK	IRRS	11.02	1.17	ss715602910	9877098–9898176
C2-08-3	C2 set	IPH	6.78	3.16	ss715600593	20295654–20975559
C2-11-1	C2 set	ΔRW	5.97	3.11	ss715609313	26075475–26169302
C2-13-1	C2-US	ΔSW	6.5	9.19	ss715617255	13389672–13550863
C2-13-2	C2-GRIN	IRRS	6.04	0.21	ss715616837	15952204–16017061
C2-13-3	C2-SK	ΔRW	5.96	10.37	ss715615656	18315025–18531998
		ΔSW	6.58	13.11	ss715615656	
C2-13-4	C2-US	IRRS	5.44	5.03	ss715614099	19599094–19614217
C2-13-5^g^	C2 set	IPH	5.51	2.07	ss715614543	28001686–28051574
C2-13-6^h^	C2 set	IRW	6.21	3.45	ss715614993	30502735–30618405
C2-13-7^i^	C2-US	IRW	6.82	11.2	ss715615007	30646059–30654291
C2-14-1	C2-SK	IRW	7.84	4.72	ss715618005	2131853–2153133
C2-15-1	C2-SK	IRRS	5.72	1.01	ss715621545	2952387–3182673
C2-16-1	C2-SK	IRRS	17.2	7.52	ss715623885	27437538–27660360
C2-17-1	C2 set	ΔRW	7.32	4.54	ss715626781	33515060–33574931
C2-17-2	C2-SK	IRW	7.18	2.38	ss715627019	36398362–36411792
C2-18-1	C2 set	IPH	5.51	2.21	ss715630573	347317–441123

^a^, Name of the QDRL in which “C2” indicates where the trait was mapped in the assay with isolate C2.S1, followed by “-##”in which the two digit number represents the chromosome to which the QDRL is located, and “-#” in which the number represents the order the QDRL are found on the chromosome.

^b^, SK, South Korea; US, North Central US; GRIN, full diversity of germplasm collection.

^c^, IRRS, inoculated root rot score; IRW, inoculated root weight; ISW, inoculated shoot weight; IPH, inoculated plant height; ΔRW, change in root weight; ΔSW, change in shoot weight; NRW, non-inoculated root weight; NSW, non-inoculated shoot weight; NPH, non-inoculated plant height

^d^, PVE, prevent variation explained.

^e^, Marker identified as associated with trait in GWA analysis

^f^, Basepair position flanking QDRL determined by linkage.

^g^, QDRL within 500kb of OH-13-1.

^h^, QDRL within 500kb of OH-13-2, OH-13-3, OH-13-4, OH-13-5.

^i^, QDRL within 500kb of OH-13-4, OH-13-5.

**Table 4:** Quantitative disease resistance loci (QDRL) mapped in the OH set.

Name^a^	Pop^b^	Trait^c^	-log_10_(p)	PVE^d^	Marker^e^	Flanking positions^f^
OH-02-1	OH set	ΔSW	5.58	2.69	ss715582345	38935468–39002760
OH-02-2	OH-SK	IRRS	5.95	6.36	ss715582359	39090899–39126047
OH-03-1	OH-SK	IRW	8.36	4.76	ss715586915	6074620–6378977
		ISW	5.46	4.67		
OH-03-2	OH-SK	IRRS	6.63	8.47	ss715586985	7872384–8252615
OH-03-3	OH-SK	ΔSW	10.04	8.58	ss715586306	42506684–42517511
OH-05-1	OH-GRIN	IPH	7.08	7.43	ss715591382	36972839–37035513
OH-06-1	OH-US	IPH	5.48	7.79	ss715595238	50603738–50852296
		IRRS	5.91	8.71	ss715595238	
OH-10-1	OH-SK	IRW	5.32	4.19	ss715605487	998272–1022198
OH-10-2	OH-SK	ISW	6.13	4.45	ss715606258	33174697–33499135
OH-11-1	OH-GRIN	IRW	6.61	8.88	ss715610747	4563815–4586936
OH-11-2	OH-GRIN	ΔSW	5.47	12.1	ss715610923	5898743–5962759
OH-12-1	OH-SK	ΔRW	5.94	10.73	ss715611695	1804514–1844191
OH-13-1^g^	OH-GRIN	IRRS	5.62	6.75	ss715614516	27874365–27896769
OH-13-2^h^	OH set	IRW	8.06	2.61	ss715614895	29971253–30065880
OH-13-3^h^	OH-US	IRW	5.87	8.02	ss715614914	30086805–30144416
OH-13-4^h^^,^^i^	OH set	IRRS	5.98	2.57	ss715614952	30291675–30301385
		IRW	7.44	2.28	ss715614952	
OH-13-5^h^^,^^i^	OH set	ΔRW	5.75	3.06	ss715615020	30667266–30700217
OH-18-1	OH-US	ISW	4.83	7.19	ss715629906	2128180–2155661
OH-18-2	OH set	IRRS	5.91	2.93	ss715632090	54737619–54774006
OH-20-1	OH-GRIN	IPH	5.41	8.89	ss715636836	1724545–1897580
OH-20-2	OH-GRIN	IPH	5.55	7.72	ss715638609	45279755–45458003

^a^, Name of the QDRL in which “OH” that indicates where the trait was mapped in the assay with isolate OH.121, is followed by “-##”in which the two digit number represents the chromosome to which the QDRL is located, and “-#” in which the number represents the order the QDRL are found on the chromosome.

^b^, SK, South Korea; US, North Central US; GRIN, full diversity of germplasm collection.

^c^, IRRS, inoculated root rot score; IRW, inoculated root weight; ISW, inoculated shoot weight; IPH, inoculated plant height; ΔRW, change in root weight; ΔSW, change in shoot weight; NRW, non-inoculated root weight; NSW, non-inoculated shoot weight; NPH, non-inoculated plant height

^d^, PVE, prevent variation explained.

^e^, Marker identified as associated with trait in GWA analysis

^f^, Basepair position flanking QDRL determined by linkage.

^g^, QDRL within 500kb of C2-13-5.

^h^, QDRL within 500kb of C2-13-6.

^i^, QDRL within 500kb of C2-13-7.

GWA analyses of the C2 set, as well as C2-SK, C2-US, and C2-GRIN populations resulted in a total of 24 significant marker-trait associations, representing 23 QDRL as defined by haplotype blocks. These 23 QDRL were comprised of 12 QDRL identified from the C2-SK population and distributed to ten chromosomes, three QDRL on Chr13 identified in the C2-US population, one QDRL on Chr13 identified in the C2-GRIN population, and seven QDRL identified from the C2 set and distributed to five chromosomes (Table 3, Fig 2). Three quantitative trait loci (QTL) associated with non-inoculated traits were identified from the GRIN population and located on Chr02. None of the QTL coincided with the identified QDRL (S7 Table, S9 Fig).

GWA analyses in the OH set, as well as OH-SK, OH-US, and OH-GRIN populations resulted in a total of 24 marker-trait associations, representing 21 QDRL as defined by haplotype blocks. These 21 QDRL were comprised of seven QDRL identified from the OH-SK population distributed to four chromosomes; three QDRL identified from the OH-US population and distributed to three chromosomes; six QDRL identified in the OH-GRIN population distributed to four chromosomes; and five QDRL identified using the OH set distributed three chromosomes. GWA analyses for non-inoculated traits identified 13 QTL distributed on 12 chromosomes (S10 Fig, S7 Table). On Chr05, a QTL for NPH was coincident with QDRL OH-5-1 for IPH, indicating that this locus may control plant architecture rather than a QDRL, *per se*.

### Comparisons of GWA analyses between the C2 and OH sets

GWA analyses in the C2 and OH sets and the populations derived from these sets resulted in the identification of 44 QDRL on 16 chromosomes; with 23 QDRL distributed to 12 chromosomes identified in the C2 set and 21 QDRL distributed to 10 chromosomes identified in OH set. Little overlap was observed between the results of the OH set and C2 set. Only QDRL C13-5, C13-6, C13-7, O13-4, and O13-5 (Tables 3 and 4; Figs 2 and 3) were identified in similar regions (<500 kb apart) between OH and C2 sets.

**Fig 4:**
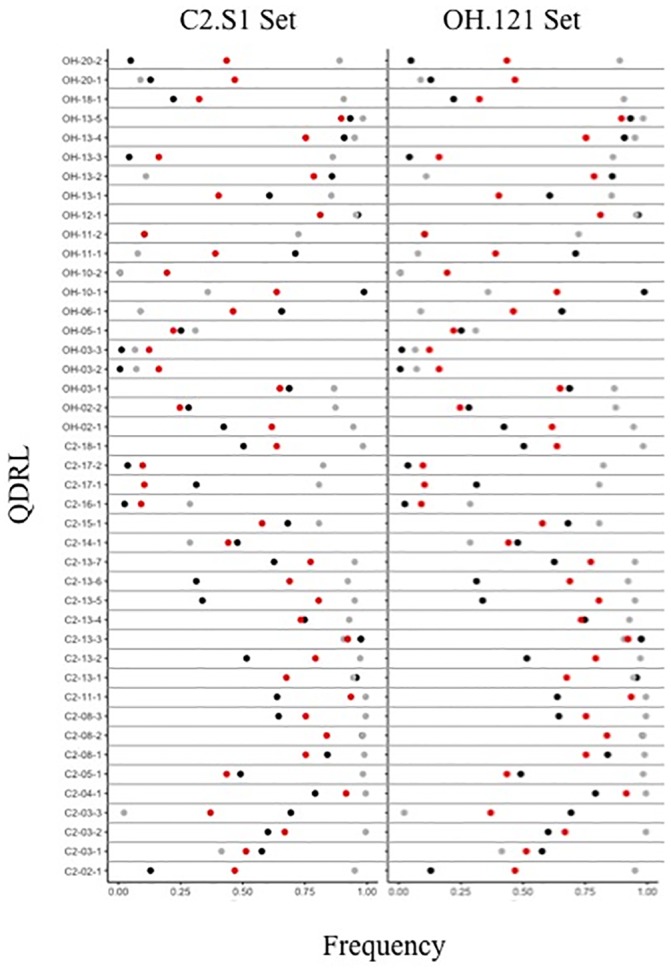
Allele frequencies of SNPs in the combined US, SK, and GRIN populations in the C2 and OH sets of PIs. The frequency of SNP alleles was calculated by adding the total number of individuals with the first allele (first in alphabetical order of SNP) divided by the total number of individuals in the population. The US population is represented by grey dots, the SK population by black dots and the GRIN population with red dots. A χ^2^ test was completed to test for significant differences in allele frequencies. All allele frequencies were significantly different (P value < 0.01).

Within a given set (C2 or OH), different QDRL were detected depending on the population. For example, the C2-SK population identified different QDRL than the C2-US and C2-GRIN. Similar differences were also observed among the QDRL identified from the populations within the OH set. In this case, the populations within a given set are structured and allele frequencies are expected to contribute to the differences in the QDRL that were detected. Indeed, there were significant differences in allele frequencies between populations (P value < 0.01) at all identified QDRL (Fig 4).

**Fig 5:**
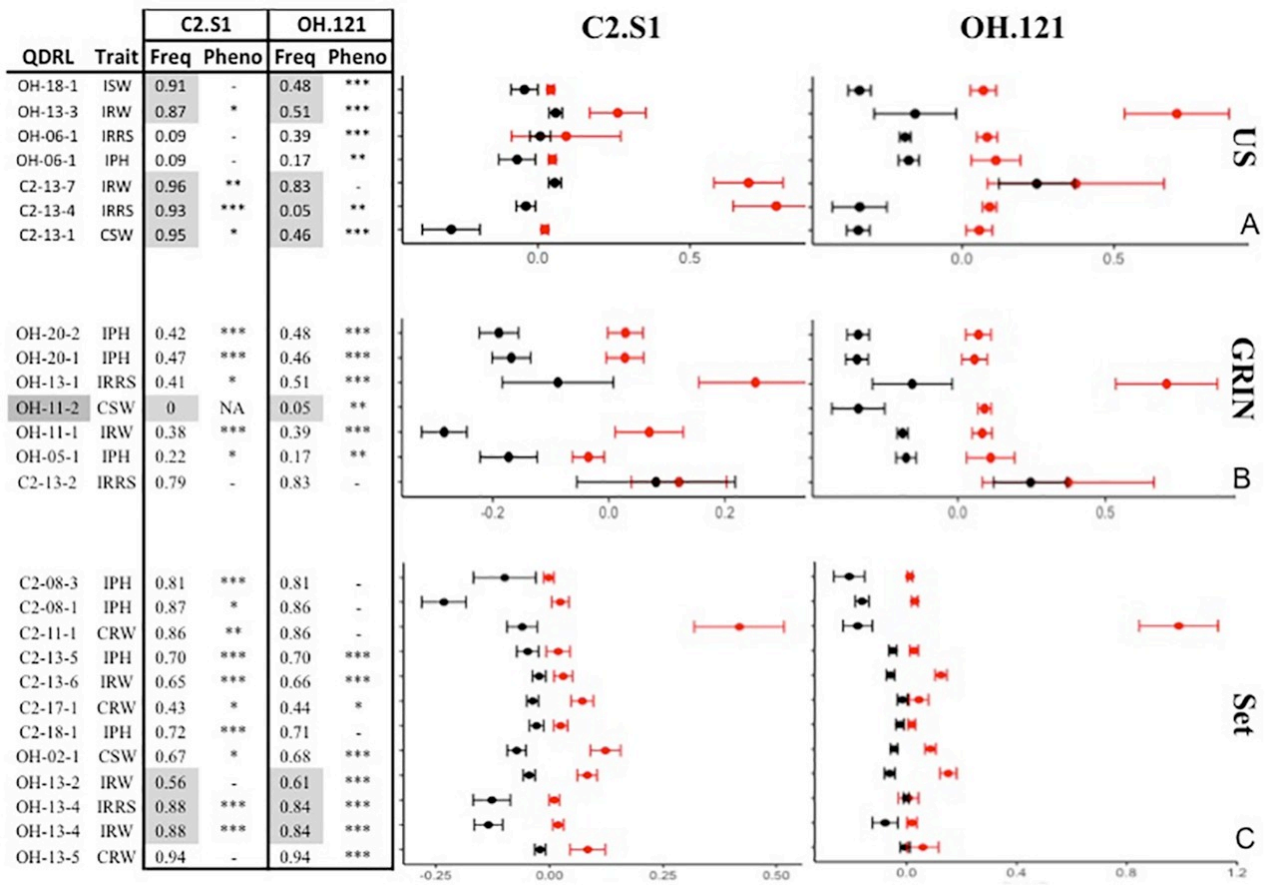
Allele frequencies and average BLUP values of PIs with resistant and susceptible alleles at QDRL identified in GWA analyses in the US, GRIN, and set populations. The frequency of the resistant allele is indicated in the “Freq” column. Significantly different allele frequencies (χ^2^ test, α = 0.05) and instances where one population is monomorphic were indicated with grey shading. For resistant (red) and susceptible (black) alleles of each QDRL, means and standard errors were calculated from the population in which the significant association was identified and are indicated with points and bars, respectively. P values from t-test are presented in the “Pheno” column with P value < 0.05 indicated with *, < 0.01 indicated with ** and < 0.001 indicated with ***. In order to consistently have the resistance indicated as a higher BLUP value, BLUP values for inoculated root rot score (IRRS) were multiplied by “-1”. Eight QDRL had a greater distribution of BLUP values and were displayed on a separate chart to aid readability. (A) US population allele frequency and average BLUP values. (B) GRIN population allele frequency and average BLUP values. (C) Set population allele frequency and BLUP values.

There were also some differences in allele frequencies from the corresponding populations within the C2 and OH sets (i.e., C2-US vs. OH-US, C2-SK vs. OH-SK and C2-GRIN vs. OH-GRIN), despite sampling the same population-groups from the population structure analysis (Fig 1). While the majority of allele frequencies were very similar and not significantly different, eight alleles were significantly different between populations (Fig 5) with five significant differences in allele frequencies between the US populations and three significant differences between the C2 set and OH set. The ability to detect QDRL is affected by allele frequency [79] and could contribute to the differences in identified QDRL.

### Corroboration of the identified QDRL and direction of allelic effects

**Fig 6:**
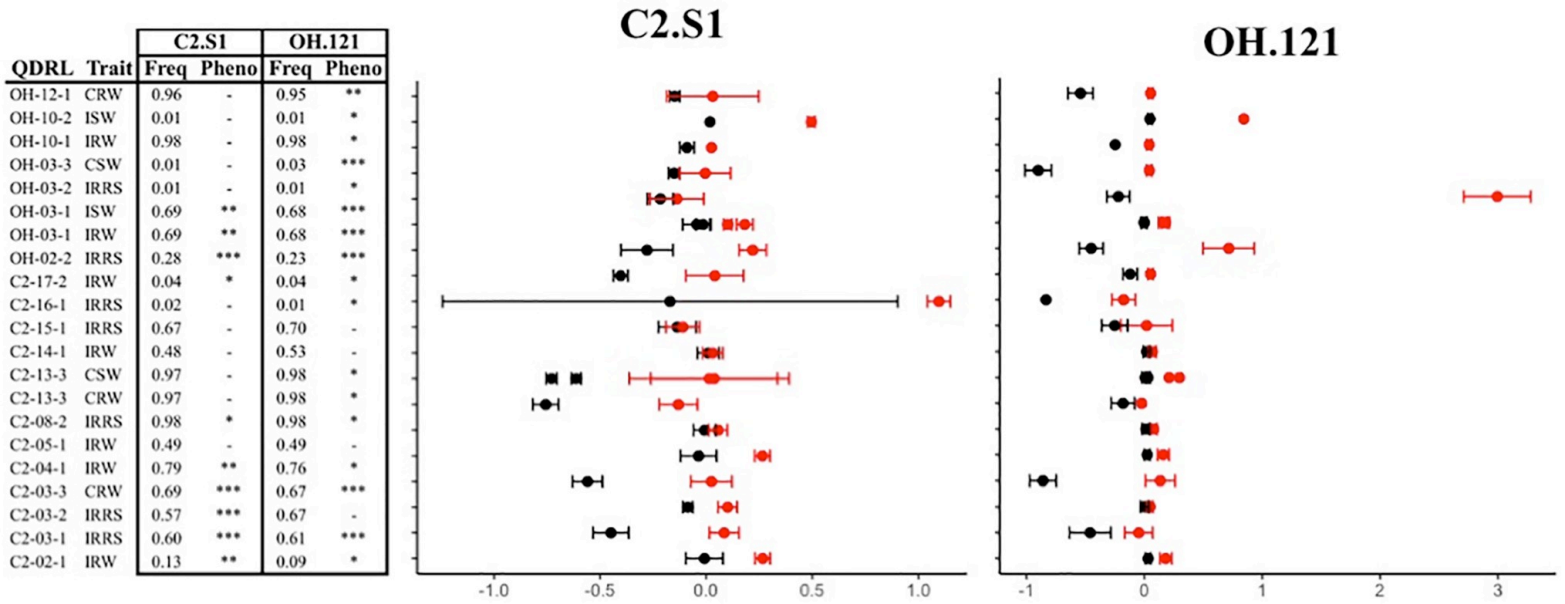
Allele frequencies and average BLUP values of PIs with resistant and susceptible alleles at the QDRL identified in GWA analyses in the C2-SK and OH-SK populations. The frequency of the resistant allele is indicated in the “Freq” column, none were significantly different (χ^2^ test, α = 0.05). For resistant (red) and susceptible (black) alleles of each QDRL, means and standard errors were calculated from the population in which the significant association was identified and is indicated with points and bars, respectively. P values from t-test are presented in the “Pheno” column with P value < 0.05 indicated with *, < 0.01 indicated with ** and < 0.001 indicated with ***. In order to consistently have the resistance indicated as a higher BLUP value, BLUP values for inoculated root rot score (IRRS) were multiplied by “-1”. Eight QDRL had a greater distribution of BLUP values and were displayed on a separate chart to aid readability. Significantly different allele frequencies were identified with a χ^2^ test and are indicated with grey shading.

There was little overlap in the QDRL identified in the C2 set and OH sets. However, single marker analyses, comparing the phenotypic means of PIs with the resistant or susceptible allele at the identified QDRL, verified the direction and allelic effect in relevant traits (Figs 5 and 6). At 43 of 44 QDRL, PIs with the resistance allele had BLUP values indicating a higher level of resistance than PIs with a susceptible allele in both the population in which the QDRL was identified and the corresponding population assayed with the alternative isolate (Figs 5 and 6). The only exception was the significant marker for OH-11-2, which was not polymorphic in the C2 set.

The QDRL markers were often significantly associated with QDR towards either OH.121 or C2.S1 in their respective populations. Using single marker analyses, most markers (37 of 44) had a significant marker effect for the isolate, population, and trait in which the association was originally identified by GWA analysis. More surprising is that a large number (26 of 44) of the single marker tests also had significant effects for QDR to the alternate isolate in the corresponding population. Thus, in addition to the direction of allelic effects being the same between the two isolates and their corresponding populations, these allelic effects were often significant based on single marker analyses.

For most of the corresponding populations, the significant markers had a similar allele frequency in both populations (e.g. C2-GRIN and OH-GRIN) (Figs 5 and 6). However, for nine QDRL, the allele frequency was significantly different between populations or monomorphic in one population. Of the 18 QDRL that were not confirmed in the relevant population for resistance to the alternate isolate, one was monomorphic and three had significantly different allele frequencies in the relevant population corresponding to the alternative isolate. In these instances, differences in the marker effect may be due to population sampling rather than isolate or environment.

**Fig 7:**
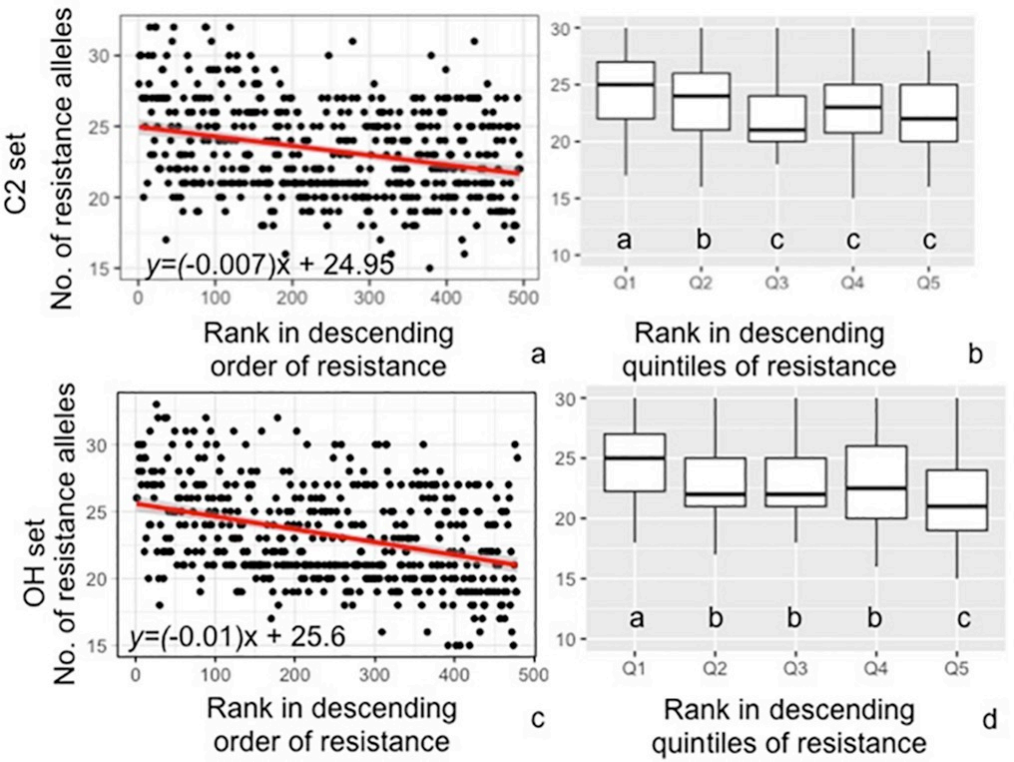
Relationship between PI QDR level and the number of resistance alleles at the identified QDRL. The total number of resistant alleles from all QDRL was calculated for all PI in the C2 set (a,b) and OH set (c,d). An overall resistance score was calculated by ranking each PI on the basis of a summed rank of the PI for each inoculated trait. The linear relationship between rank and resistant allele number was calculated in the C2 set (a) and OH set (c). The average rank and resistant allele number was calculated in quintiles in the C2 set (b) and OH set (d). Different letters indicate significant differences in rank between quintiles as determined by Fisher’s protected least significant difference (P value < 0.05).

In both the C2 and OH sets, there was a significant correlation (P value <0.001) between the number of resistant alleles and an overall resistance rank (Fig 7a and 7c). In the C2 set, the most resistant quintile averaged 25.2 resistance alleles (of 44), significantly higher than the other quantiles (Fig 7b). The OH set displayed a similar trend with the most resistant quintile averaging 25.8 resistance alleles, significantly more resistance alleles than the other quintiles (Fig 7d).

### Meta-QDRL analysis

Comparison to previously identified QDRL would provide further evidence to validate our findings as well as distinguish the novel QDRL identified in this study. While over a hundred QDRL for resistance to *P*. *sojae* have been previously identified from bi-parental mapping studies, many were identified in only a single study. Thus, in order to focus on the robust QDRL, we completed a meta-analysis of QDRL.

In addition to four GWA studies (S8 Table), 16 biparental mapping studies have also been completed (S2 Table), identifying 143 QDRL positions (77 positions on Soybase) [61]. The studies were filtered such that only a single mapping study from the same biparental cross and generation is represented and studies prior to 2010 were removed. The remaining 11 studies describe 114 QDRL and were included in this meta-analysis. The meta-analysis of the QDRL consolidated these results into 22 robust meta-QDRL distributed across ten chromosomes (Table 5). Based on the reference genome (Wms.82.a2), the meta-QDRL are associated with an average physical region spanning 5547kb (Table 5).

**Table 5:** *Phytophthora sojae* QDRL identified in meta-analyses of previously completed biparental mapping studies.

Name^a^	Flanking Markers^b^	Flanking Positions^c^
m01-1	BARC-035199-07136—BARC_2.0_Gm01_3237203	1031156–3237203
m01-2	BARC_2.0_Gm01_3342559—Sat_414	3342559–52336556
m02-1	BARC-013499-00502—BARC-063263-18286	2411666–4581169
m02-2	BARC-047945-10443—BARC-043983-08572	15054401–15694465
m04-1	BARC-030751-06938—BARC-019015-03051	2062813–5241170
m06-1	BARC-05997-16280—ss1235978364	5449370–7564115
m06-2	BARC-053603-11920—Sat316	47944572–48016485
m06-3	BARC-063259-18282—ss1235977679	11363772–11755372
m08-1	BARC-032503-08989—BARC-057257-14650	7780999–9484485
m08-2	BARC-017983-02492—BARC-040893-07862	10473841–11762085
m08-3	BARC-059367-15768—BARC-050015-09290	40735744–42086643
m09-1	BARC-064511-18708—Satt417	11755372–18102265
m13-1	Sat_297—Sat_298	19951364–26976064
m13-2	BARC-018521-02928—Satt335	31794058–31831950
m13-3	AW756935—BARC-016585-02149	41606169–44344337
m15-1	Satt411—BARC-008231-00112	2517404–3964389
m15-2	BARC_2.0_Gm15_6841404—BARC_2.0_Gm15_9983279	6841404–9983279
m16-1	BARC_2.0_Gm16_298020—BARC_2.0_Gm16_759723	298020–759723
m16-2	BARC_2.0_Gm16_27864182—BARC_2.0_Gm16_58887013	58887013–27864182
m18-1	BARC-020839-03962—ss1235985359	981868–2396395
m18-2	Sat_141—BARC-005806-00273	2418920–3276029
m18-3	BARC_2.0_Gm18_5151897—Satt325	5151897–8587948

^a^, The name of the meta-QTL, with the m prefix indicating that it is a meta-QDRL, followed by “-##”in which the two digit number represents the chromosome to which the QDRL is located, and “-#” in which the number represents the order the QDRL are found on the chromosome.

^b^, Left and right flanking markers determined by meta-QDRL analyses

^c^, basepair position of flanking markers QDRL

### Colocalization between previous research and current results

We identified regions most often associated with resistance by comparing current results with the four previous GWA analyses for QDR to *P*. *sojae* [55–59], as well as the meta-QDRL (Table 5). QDRL C2-02-1, C2-03-3, C2-13-7, and C2-17-2 were < 500kb from regions identified in previous GWA analyses, adding evidence to these regions being important in QDR. No such colocalizations were identified for QDRL from the OH set or populations (Table 6).

**Table 6:** Summary of colocalizations of identified QDRL with previously identified QDRL, *Rps-*genes, or other pathogens.

QDRL	Other QDRL	*Rps-*genes	Other Pathogens^a^	Citation(s)
C2-02-1	Satt634^b^	*RpsZS18*		[57]/S1 Table
C2-03-1		*Rps1*		S1 Table
C2-03-2		*Rps1*		S1 Table
C2-03-3	ss715585728			[55]
OH-03-3			SCN	[80]
C2-04-1	14–7^c^			S2 Table
OH-05-1			SDS	[81,82]
OH-06-1			SDS	[83–85]
C2-08-2	m08-1			Table 5
C2-08-3			Sclerotinia & SDS	[86,87]
C2-11-1			SCN	[82]
C2-13-2	1–1, 2–1, 4–1, 3–1, 9–2^c^			S2 Table
C2-13-3	1–1, 2–1, 4–1, 3–1, 9–2^c^			S2 Table
C2-13-4	m13-1			Table 5
C2-13-5		*Rps3*		S1 Table
C2-13-6		*Rps3*		S1 Table
C2-13-7	ss715615031^b^	*Rps3*		[55]
OH-13-1		*Rps3*		S1 Table
OH-13-2		*Rps3*		S1 Table
OH-13-3		*Rps3*		S1 Table
OH-13-4		*Rps3*		S1 Table
OH-13-5		*Rps3*		S1 Table
C2-15-1	m15-1			Table 5
C2-16-1	m16-2			Table 5
C2-17-1		*Rps10*		S1 Table
C2-17-2	Satt301^b^	*Rps10*		[57]/S1 Table
OH-18-1	m18-1			Table 5
OH-18-2		*Rps6*		S1 Table
OH-20-1			SDS	[82]
OH-20-2			SCN	[80]

^a^, identified resistance loci to other pathogens when no *P*. *sojae* resistance colocalized with identified QDRL. Colocalization with other pathosystems was not searched if colocalization was identified with other *P*. *sojae* resistance.

^b^, Significant marker referenced in GWA analysis.

^c^, Name of QDRL as stored in Soybase.

Five QDRL from our GWA analyses are near (within 500kb) or overlap with meta-QDRL; m08-1 near C2-08-02 (C2-SK), C2-13-4 (C2-US) near m13-1, C2-15-1 (C2-SK) near m15-1, C2-16-1 (C2-SK) near m16-2, and OH-18-1 (OH-US) near both m18-1 and m18-2 (Figs 2 and 3). Because specific criteria were used to determine which QDRL mapping studies to include in the meta-analysis, a number of previously reported QDRL were not included. The QDRL C2-04-1, C2-13-2, and C2-13-3 co-localized with previously reported “non”-meta-QDRL because these QDRL were either not included in the meta-analysis or the QDRL were merged into meta-QDRL at a slightly different genetic position (Table 6).

In addition to the 12 QDRL from our GWA analyses which overlapped with previously identified QDRL, fourteen of the QDRL identified in the present study were within or < 500kb from reported *Rps*-gene locations. QDRL C2-02-1 co-localized with *RpsZS18*. C2-03-1 and C2-03-2 were near the *Rps1* region. We identified QDRL C2-13-5, C2-13-6, and C2-13-7 within the *Rps3* region, and the two QDRL identified on Chr17, C2-17-1 and C2-17-2, were within the *Rps10* region. In the PIs phenotyped with OH.121, OH-18-2 was near *Rps6*; OH-13-1, OH-13-2, OH-13-3, OH-13-4, and O13-5 were all near *Rps3* on Chr13 (Table 6).

Some QDRL identified in this study were novel in their association towards *P*. *sojae* disease resistance, but were in regions previously associated with resistance to other soybean pathogens and pests. QDRL OH-03-3 is in a region associated with resistance to whiteflies and soybean cyst nematode (SCN) [80]. QDRL OH-05-1 is in a region associated with resistance to the toxins produced by *Fusarium virguliforme* [81,82]. QDRL OH-06-1 is near the position of a QDRL associated with resistance to sudden death syndrome (SDS) [83–85]. QDRL C2-08-3 was within regions associated with Sclerotinia and SDS resistance [86,87]. QDRL C2-11-1 is near QDRL for SCN [88]. QDRL OH-20-1 is in a region associated with QDR to SDS [82]. OH-20-2 in a region associated with QDR to SCN [80] (Table 6). Finally, the remaining 14 QDRL are in regions not previously associated with resistance to fungal, viral, or nematode pathogens based on information available in Soybase [61] (March 1^st^, 2019).

## Discussion

### A large and diverse population of soybean accessions led to the identification of 44 QDRL

In this study, six traits for QDR were measured for PIs sampled from the USDA collection, representing germplasm from the US, the Republic of Korea, as well as PIs that were sampled without geographic consideration. PIs were phenotyped following inoculation with either *P*. *sojae* isolate C2.S1 or OH.121, forming the two sets of 495 PIs in the C2 set and 478 PIs in the OH set, respectively. Identification and comparison of QDRL for multiple isolates of *P*. *sojae* can be informative for making breeding decisions, as it is genetically complex [89], likely contributing to not only diverse virulence patterns but also other aspects of pathogenesis. Though it would have been interesting to phenotype all PIs with both isolates, a limited number of seeds were available from the USDA collection.

GWA analyses were completed separately depending on the aforementioned experimental design, and though the traits were significantly correlated, unique QDRL were identified for each measurement. Though correlated, it remains pertinent to evaluate multiple traits, as each QDRL could be functioning though different molecular mechanism or different tissue and may only be detected by measuring a specific trait [90]. Overall, 44 QDRL were identified, and though GWA analyses are expected to identify large-effect and common alleles [91], here we identified 44 small-effect QDRL explaining less than 14% of the variation, consistent with results from previous mapping studies (S2 Table).

Superficially, the GWA analyses here appear to have identified many more QDRL than previous studies. For example, GWA analyses for QDR towards *P*. *sojae* have been completed in more specific samples of germplasm and individually have identified a maximum of 16 markers associated with resistance [55]. Biparental mapping studies have also identified similar numbers of QDRL [42–48]. For example, a cross of Conrad x Hefeng 25 identified eight QDRL [44]; the cross of Conrad x Sloan identified 10 QDRL [48]; and crosses of PI 3998841 or PI 407861A with OX20-8, identified 3 and 7 QDRL, respectively [51–53]. There were a total of 16 genetic regions associated with QDR to *P*. *sojae* identified using six nested populations in a joint linkage mapping study with an average of 1.5 QDRL identified in individual populations and 6 additional QDRL detected with joint linkage analyses [53].

However, both the diversity of PIs and the completion of analyses in multiple populations likely contributed to the large number QDRL identified. Though the total number of QDRL exceeded previous GWA results, analyses within individual population are comparable to previous research. For example, 11 QDRL were identified in the C2-SK population, and seven QDRL in the OH-SK population. These results are similar to the seven QDRL identified in the study completed in SK germplasm by Schneider et al. (2016) [55]. GWA analyses in Chinese breeding lines have resulted in identifying three or one QDRLs [57,58], likewise the C2-US and OH-US populations, consisting primarily of US cultivars, resulted in the identification of 3 QDRL.

The high level of soybean diversity in the full C2 set or OH set, may allow for the detection of novel QDRL, but it also increases the population structure, and, therefore, the risk of false positives [92]. However, efforts to reduce false positives in GWA analyses were applied by using models that incorporated population structure and kinship among PIs [93]. The false-positive rate was also increased because multiple GWA analyses were completed for each population within each set (C2 and OH) of PIs. The methodology in this study was similar to that of Bandillo et al. (2015) [94], where a broad collection of PIs was initially sampled for GWA analysis, followed by analyses of four subsets from the initial collection to identify QTL for seed protein and oil content. Though testing subsets of PIs in addition to the full set does increase the false positive rate, we applied appropriate and stringent significance thresholds specific to each population based on the respective effective marker numbers [71].

### Many QDRL co-localize with previously identified loci for *P*. *sojae* resistance, as well as represent novel loci for this pathosystem

Prior to this study, 21 biparental or GWA mapping studies for QDR towards *P*. *sojae* have been completed (S2 Table). Additionally more than 30 *Rps-*genes/alleles have been identified for race-specific resistance (S1 Table). Twenty-one of the QDRL reported in this study were novel loci for this pathosystem, adding significantly to our knowledge of the genetic architecture of QDR to *P*. *sojae*. The remaining 23 QDRL colocalize with previously identified QDRL and/or *Rps*-genes. Depending on whether these co-localized resistances are in *cis*, in *trans*, or controlled by the same gene(s), the colocalization of these QDRL could have significant breeding implications.

Colocalization was identified by comparing the QDRL identified here with the 143 QDRL reported from 16 biparental mapping studies positions (S2 Table). Though the expression of QDR towards *P*. *sojae* can be dependent on environmental, methodological, and assay conditions [47], in this study we consolidated these QDRL into 22 robust meta-QDRL distributed across 10 chromosomes. In total, twelve QDRL, or 27%, colocalized with previously identified loci from GWA analyses, non-meta-QDRL for *P*. *sojae* resistance or meta-QDRL. This is similar to previous GWA studies where approximately 33% of QDRL co-localized with results from other studies [55,57,59]. Five of the 22 meta-QDRL colocalized with QDRL we identified in this study, strongly supporting the involvement of these regions in QDR to *P*. *sojae*. Though only 5 QDRL colocalized with meta-QDRL, this analysis greatly facilitated the consolidation of the growing number of identified QDRL for resistance towards *P*. *sojae*.

*Rps-*gene regions often overlap with QDRL regions [53,55]. In this study, 14 QDRL overlapped with or were within 500kb of previously reported *Rps-*gene regions. Though phenotypically distinct, the colocalization of *Rps*-genes is conspicuous, coaxing the research question: Are canonical *R*-genes functioning in a QDR? Indeed, researchers have previously proposed that QDR may be an *R*-gene-mediated response that does not confer complete resistance, resulting in race-specific QDRL [95]. While studies have previously identified QDRL that have been significant for one isolate and not other isolates [48], at this time, no significant race or isolate × QDRL interaction has been reported, which would be the hallmark of a *bona fide* race-specific QDRL. Few studies on QDR to *P*. *sojae* have specifically addressed race-specificity of QDRL, and those that have tested for race-specific QDRL did not identify any significant isolate × QDRL interaction [96]. In this study, isolate, population, as well as environment, were all confounded. Thus, we were unable to test the race or isolate-specificity of the QDRL.

### There was little overlap in QDRL identified between the C2 set and OH set

The C2 set and OH set were both comprised of US, SK and GRIN populations sampled from the USDA germplasm collection. Though the germplasm was similar, the two sets varied for isolate and the greenhouse environment, and while similar numbers of QDRL were identified in both the C2 set and OH set, there was little overlap between the QDRL identified with each isolate, among populations, or for each trait. With the exception of a region on Chr13, the QDRL identified in either the C2 set or OH set were unique to the set.

As in other host-pathosystems, QDR is often a complex trait [28]. This type of genetic complexity can be observed in the foliar disease of maize, Southern Corn Leaf Blight, where GWA analysis for resistance in the maize NAM population resulted in the identification of 32 QDRL [97]. Similarly, over 100 QDRL have been identified for the well-studied Fusarium head blight resistance in wheat [98]. Based on current and previous mapping studies for resistance to *P*. *sojae*, we can estimate that greater than 60 loci are contributing to QDR. The majority of identified QDRL toward *P*. *sojae* in soybean explain less than 15% of the variation in the population in which they were mapped. The small individual effect of each allele contributes to the complexity of the resistance phenotype itself, as well as to the genetic analysis of the trait. In fact, while many QDRL have been reported as isolate-specific or specific to an individual resistance trait or assay [47,48,53,55], it is unclear whether these specific QDRL are due to the marginal significance of small effect loci, or the functional complexity of the QDR. Interestingly, though QDRL were only identified with one isolate of *P*. *sojae*, there were significant differences in the response trait between the resistant and susceptible alleles both within the populations in which the QDRL were identified as well as within the corresponding population assayed with the alternate isolate. This provides independent corroboration for these QDRL and indicates that many, if not all, of the 44 QDRL are valid.

Among the populations that made up each isolate set, we observed unique QDRL, with no overlap in the genetic positions of QDRL identified among populations within each isolate set. As expected, we also observed unique alleles and genetic structure for each population within an isolate set. Populations were sampled from specific groups, which were determined by a population structure analysis, and possessed differences in the number of segregating markers as well as significantly different allele frequencies at QDRL. Thus, the lack of overlap between QDRL identified in each population is likely a result of some combination of differences in resistance allele polymorphism, allele frequency, and/or LD.

### Targeting multiple QDRL for different traits may provide improvement of QDR in elite cultivars

QDR towards *P*. *sojae* is thought to consist of multiple mechanisms that interact to produce a defense response [99]; as such, combining QDRL involved with different aspects of resistance could produce the highest levels of resistance. However, the question remains: How applicable are individual minor effect QDRLs, possibly only contributing to one isolate or trait, for the improvement of elite cultivars? Indeed, these are small effect QDRL, however one of the proposed benefits of QDR is durability, and minor effect QDRL are predicted to be more durable than large-effect QDRL with wide-spread deployment [91].

Specifically for US germplasm, there has been an improvement in QDR over time, but this began to plateau in the 1980s [100]. Indeed, US populations in this study had lower levels of QDR than the SK and GRIN populations. In the C2 set, only 10 of the top 50 most resistant PIs are from the C2-US population. The OH set is similar, with 11 US PIs in the top 50 most resistant PIs.

Among the most resistant PIs from the C2-US population are four Clark isolines (PI547538, PI547583, PI547476, PI547528) and one Williams isoline (PI591509) [101]. Both Clark and Williams have been reported to have moderate to high levels of QDR against *P*. *sojae* [55]. Likewise in the OH set, the most resistant lines were also from Clark (PI547496) and Williams (PI547852, PI547496, and PI634758) derivatives [105,106]. In addition, among the 11 resistant US PIs were cultivars OH FG2 (PI584470), Lonoke (P633609), and Athow (PI595926), all of which had been previously reported as moderate to highly resistant [38, 102–105]. Thus, disease assays in each of the US populations have repeatedly identified known and often well utilized sources of partial resistance to *P*. *sojae*. At a minimum, Clark and Williams have been widely incorporated into breeding programs [106]; therefore, it is possible many of the QDR alleles identified in the US populations have already been deployed in US germplasm.

Overall, there was a correlation between the number of resistant alleles and the overall resistance levels, indicating the value of stacking QDR alleles. In other pathosystems, such as rice-*Magnaporthe oryzae*, pyramiding of QDRL increased resistance [107]. Similarly, pyramiding QDR alleles for resistance to Fusarium head blight in wheat increased resistance. Interestingly, among the top 50 most resistant PIs, PIs from the US populations averaged fewer QDR alleles than the PIs from the GRIN and SK populations. Thus, there may be room for continued improvement by incorporating additional QDR alleles.

Based on this study, valuable and previously untested QDR alleles are available to soybean breeders to increase the diversity in their genetic base as they attempt to improve elite cultivars, notably from PIs originating from SK, which had high levels of QDR in this study and previous research [55,56]. The complex genetic architecture of QDR towards *P*. *sojae* indicates the importance of stacking multiple QDR alleles or a potential role for tools such as genomic selection. Efforts to introduce alleles contributing to a number of QDR traits are expected to provide the most durable and highest levels of QDR.

## Supporting information

S1 Table Positions of *Rps*-genes/alleles in the soybean genome. (XLSX)

S2 Table Published quantitative disease resistance towards *Phytophthora sojae* mapping studies. (XLSX)

S3 Table Overall ranking of resistance for plant introductions in the C2 set. PIs were ranked from most resistance to least resistant for the four inoculated traits, ΔCRW and ΔCSW, ranks were summed and re-ranked.
(XLSX)

S4 Table Overall ranking of resistance for plant introductions in the OH set. PIs were ranked from most resistance to least resistant for the four inoculated traits, ΔCRW and ΔCSW, ranks were summed and re-ranked.
(XLSX)

S5 Table FastStructure population number selection. (XLSX)

S6 Table Genetic variance and heritability in each population. (XLSX)

S7 Table Significant marker-trait associations identified for non-inoculated traits. (XLSX)

S8 Table Results of previously completed GWA analyses for *P*. *sojae* quantitative disease resistance. (XLSX)

S1 Fig Comparison of QQ-plots of different genome-wide association analysis models implemented in GAPIT. Models represented (1) GLM; general linear model, (2) MLM; mixed linear model, (3) CMLM; compressed mixed linear model, (4) MLMM; multiple linear mixed model.
(TIFF)

S2 Fig Population clustering using fastStructure indicated nine subpopulations within the SK populations. (A) Nine subpopulations identified in the C2-SK PIs. (B) Nine subpopulations identified in the OH-SK PIs (C) Model complexities from one to ten were tested identifying a plateauing marginal likelihood value at *k* = 9.
(TIFF)

S3 Fig Population clustering using fastStructure indicated six subpopulations within the US populations. (A) Six subpopulations identified in the C2-US population. (B) Six subpopulations identified in the OH-US population. (C) Model complexities from one to eight were tested identifying a plateauing marginal likelihood at *k* = 6.
(TIFF)

S4 Fig Linkage disequilibrium decay in heterochromatic and euchromatic regions and significance thresholds calculated using Meff. Linkage between markers (r^2^) as a function of physical distance (base pair) calculated in the 974 plant introductions used in GWA analyses. Significance thresholds include: ^a^, effective marker number; ^b^, calculated significance threshold [-log10(0.05/effective marker number]; ^c^, estimated effective marker density in markers per kilobases.
(TIFF)

S5 Fig Histogram distribution and qq-normality plots of the 495 PIs in C2 set used for GWA mapping. IRRS, inoculated root rot score; IRW, inoculated root weight; ISW, inoculated shoot weight; IPH, inoculated plant height; ΔRW, change in root weight; ΔSW, change in shoot weight; NRW, non-inoculated root weight; NSW, non-inoculated shoot weight; NPH, non-inoculated plant height.
(TIFF)

S6 Fig Comparison of BLUP values between the US, SK, and GRIN populations in the C2 set. The GRIN population (red) the SK population (white) boxplots, and the US population (grey) populations are represented for all nine traits: IRRS, inoculated root rot score; IRW, inoculated root weight; ISW, inoculated shoot weight; IPH, inoculated plant height; ΔRW, change in root weight; ΔSW, change in shoot weight; NRW, non-inoculated root weight; NSW, non-inoculated shoot weight; NPH, non-inoculated plant height. Significantly differences in population determined using Fisher’s protected LSDs (P value <0.05) are indicated with letters.
(TIFF)

S7 Fig Histogram distribution and qq-normality plots of the 478 PIs in OH set used for GWA mapping. IRRS, inoculated root rot score; IRW, inoculated root weight; ISW, inoculated shoot weight; IPH, inoculated plant height; ΔRW, change in root weight; ΔSW, change in shoot weight; NRW, non-inoculated root weight; NSW, non-inoculated shoot weight; NPH, non-inoculated plant height.
(TIFF)

S8 Fig Comparison of BLUP values between the US, SK, and GRIN populations in the OH Set. The GRIN population (red) the SK population (white) boxplots, and the US population (grey) populations are represented for all nine traits: IRRS, inoculated root rot score; IRW, inoculated root weight; ISW, inoculated shoot weight; IPH, inoculated plant height; ΔRW, change in root weight; ΔSW, change in shoot weight; NRW, non-inoculated root weight; NSW, non-inoculated shoot weight; NPH, non-inoculated plant height. Significantly differences in population were tested using Fisher’s protected LSDs (P value <0.05) are indicated with letters.
(TIFF)

S9 Fig Manhattan plot results of non-inoculated traits in the C2 set. Population the marker-trait association was identified in via the shape of the significant marker. The specific trait is identified by the color of the marker. Significance thresholds calculated using SimpleM are displayed for the C2 set, and the C2-US Population.
(TIFF)

S10 Fig Manhattan plot results of non-inoculated traits in the OH set. The figure shows which population the marker-trait association was identified in via the shape of the significant marker. The specific trait is identified by the color of the marker. Significance thresholds calculated using SimpleM are displayed for the OH set, and the OH-US Population.
(TIFF)
