# Supplementary figures and images for: Genome-wide association analyses of quantitative disease resistance in diverse sets of soybean [Glycine max (L.) Merr.] plant introductions
Source: PLoS One. 2020 Mar 20;15(3):e0227710. doi: 10.1371/journal.pone.0227710 (PMC7083333; doi:10.1371/journal.pone.0227710)

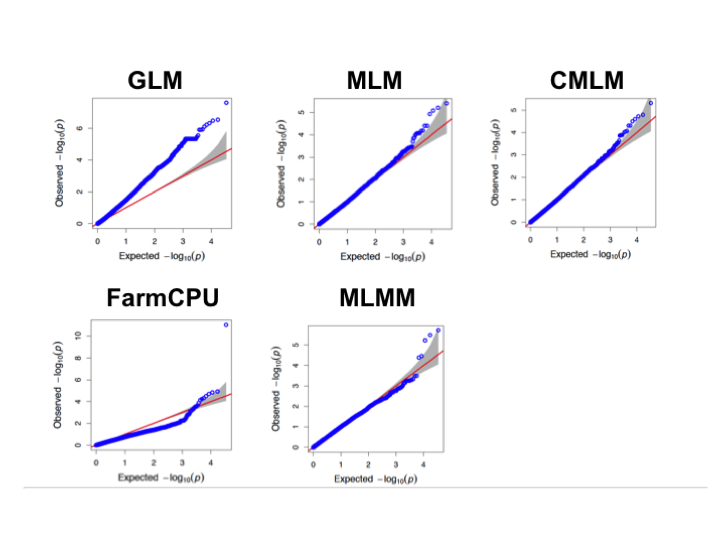

Supplement: S1 Fig — Models represented (1) GLM; general linear model, (2) MLM; mixed linear model, (3) CMLM; compressed mixed linear model, (4) MLMM; multiple linear mixed model. (TIFF) [file pone.0227710.s009.tiff]

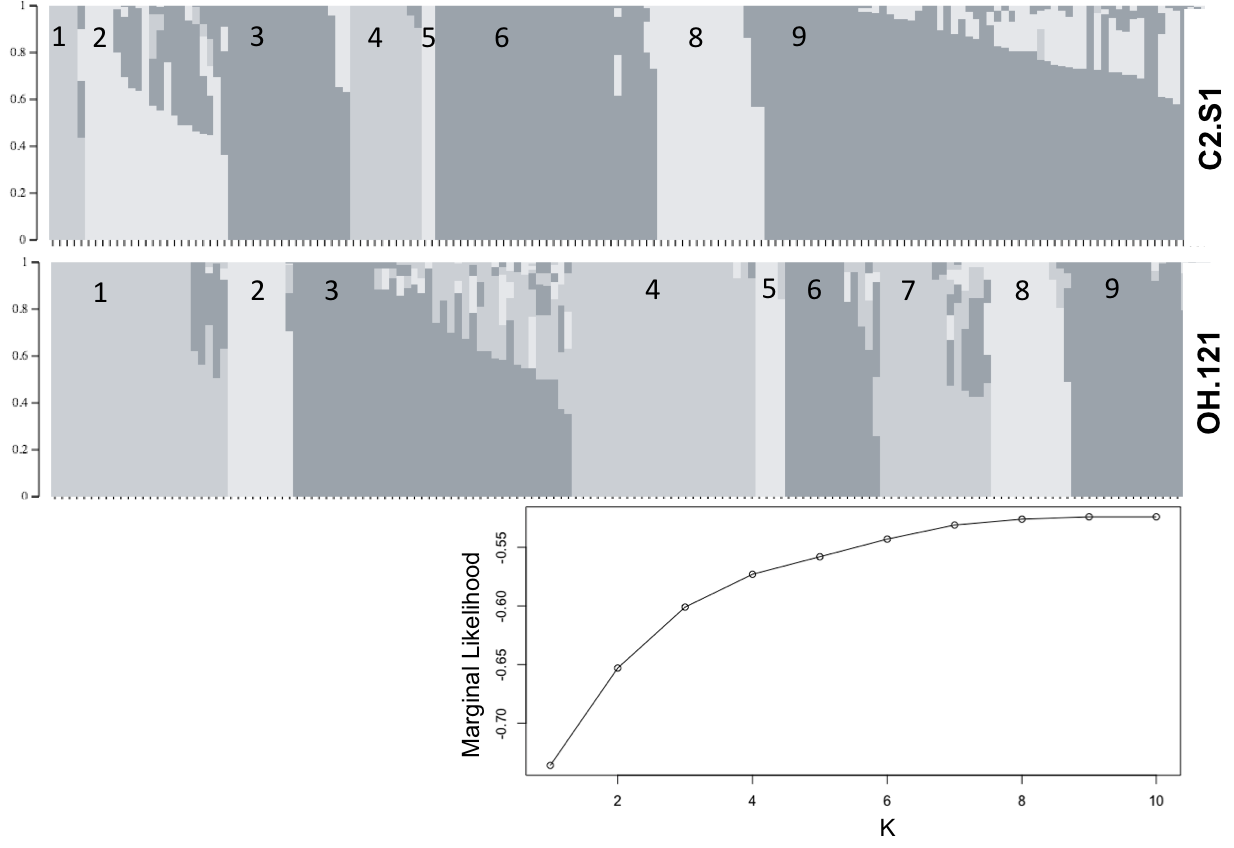

Supplement: S2 Fig — (A) Nine subpopulations identified in the C2-SK PIs. (B) Nine subpopulations identified in the OH-SK PIs (C) Model complexities from one to ten were tested identifying a plateauing marginal likelihood value at k = 9. (TIFF) [file pone.0227710.s010.tiff]

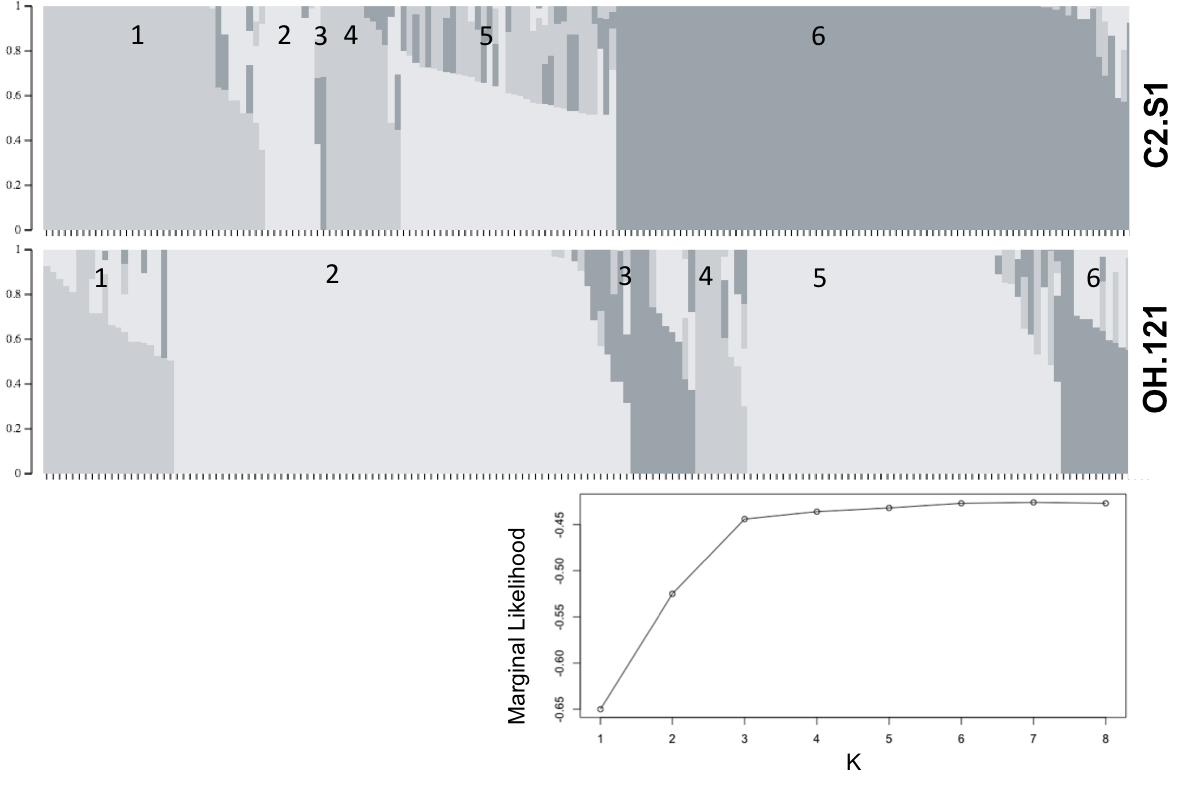

Supplement: S3 Fig — (A) Six subpopulations identified in the C2-US population. (B) Six subpopulations identified in the OH-US population. (C) Model complexities from one to eight were tested identifying a plateauing marginal likelihood at k = 6. (TIFF) [file pone.0227710.s011.tiff]

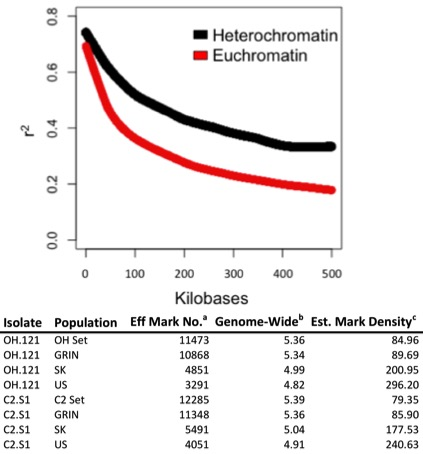

Supplement: S4 Fig — Linkage between markers (r2) as a function of physical distance (base pair) calculated in the 974 plant introductions used in GWA analyses. Significance thresholds include: a, effective marker number; b, calculated significance threshold [-log10(0.05/effective marker number]; c, estimated effective marker density in markers per kilobases. (TIFF) [file pone.0227710.s012.tiff]

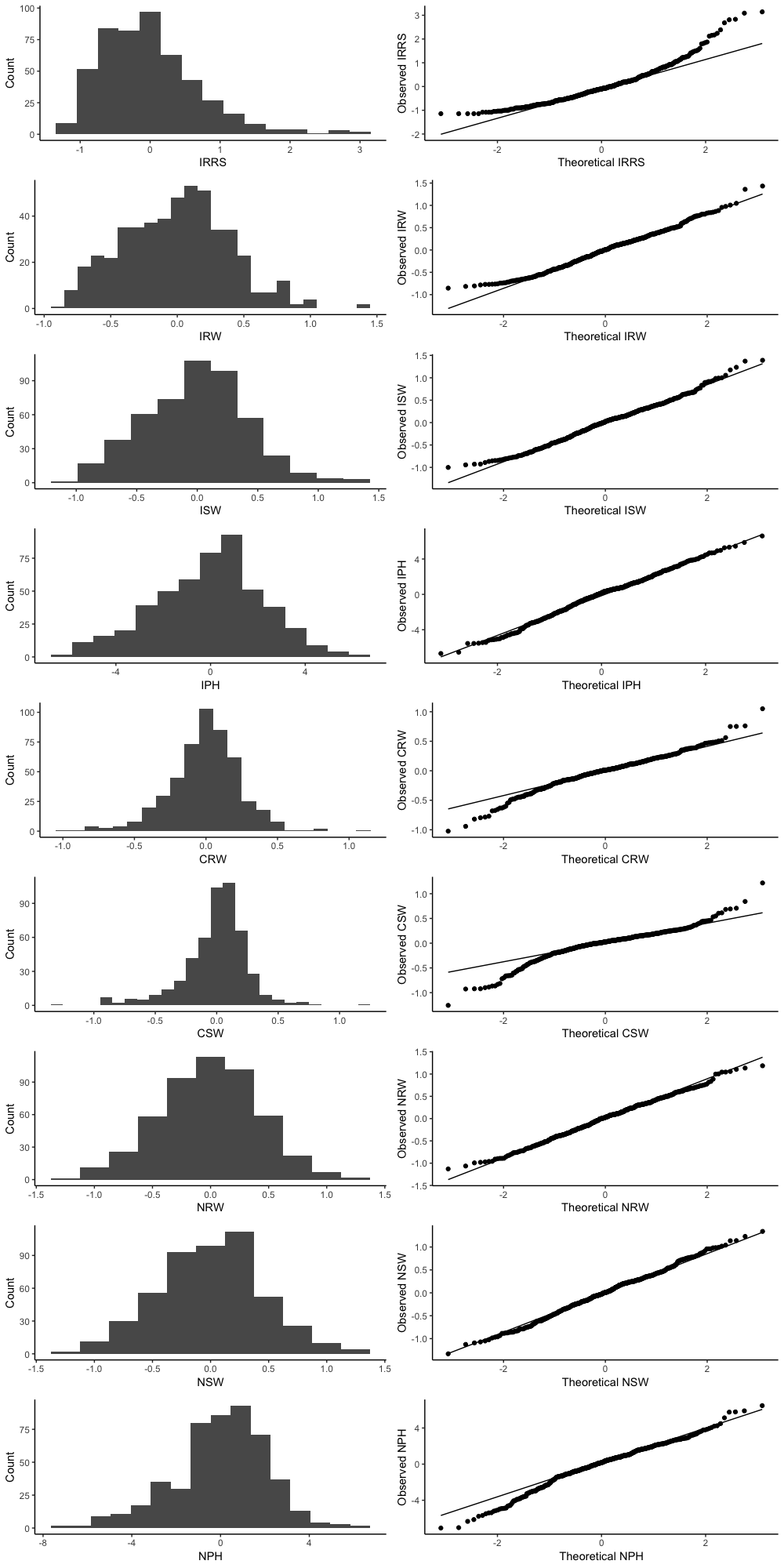

Supplement: S5 Fig — IRRS, inoculated root rot score; IRW, inoculated root weight; ISW, inoculated shoot weight; IPH, inoculated plant height; ΔRW, change in root weight; ΔSW, change in shoot weight; NRW, non-inoculated root weight; NSW, non-inoculated shoot weight; NPH, non-inoculated plant height. (TIFF) [file pone.0227710.s013.tiff]

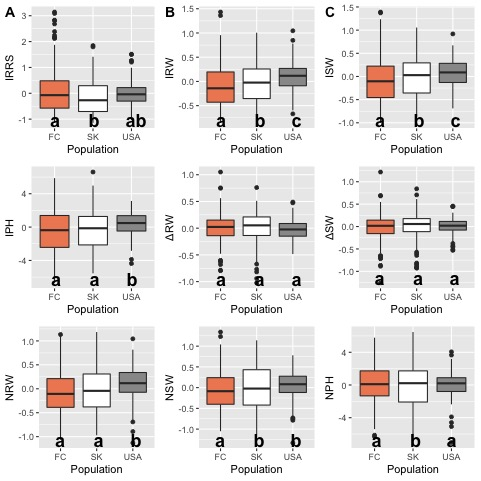

Supplement: S6 Fig — The GRIN population (red) the SK population (white) boxplots, and the US population (grey) populations are represented for all nine traits: IRRS, inoculated root rot score; IRW, inoculated root weight; ISW, inoculated shoot weight; IPH, inoculated plant height; ΔRW, change in root weight; ΔSW, change in shoot weight; NRW, non-inoculated root weight; NSW, non-inoculated shoot weight; NPH, non-inoculated plant height. Significantly differences in population determined using Fisher’s protected LSDs (P value <0.05) are indicated with letters. (TIFF) [file pone.0227710.s014.tiff]

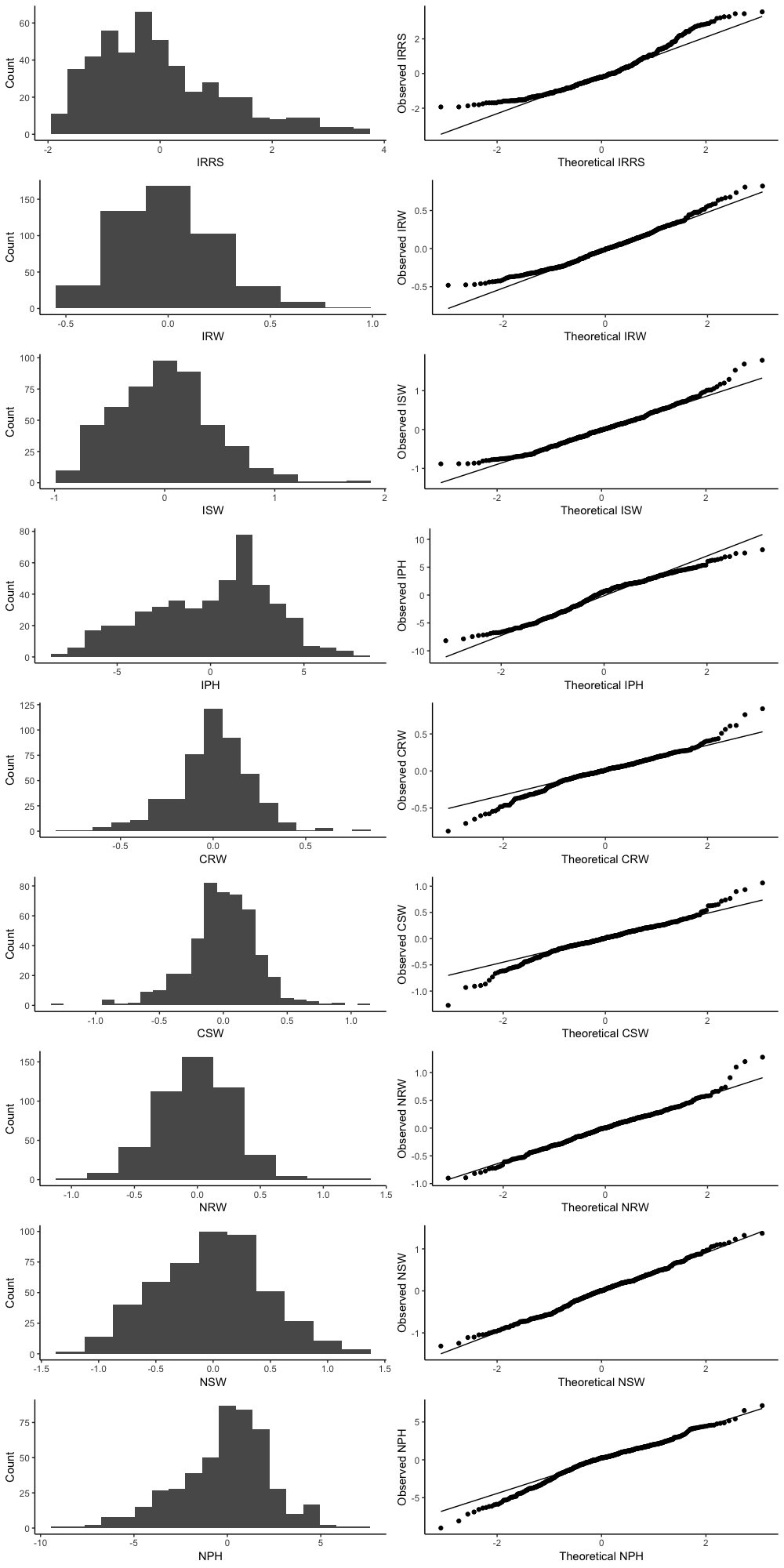

Supplement: S7 Fig — IRRS, inoculated root rot score; IRW, inoculated root weight; ISW, inoculated shoot weight; IPH, inoculated plant height; ΔRW, change in root weight; ΔSW, change in shoot weight; NRW, non-inoculated root weight; NSW, non-inoculated shoot weight; NPH, non-inoculated plant height. (TIFF) [file pone.0227710.s015.tiff]

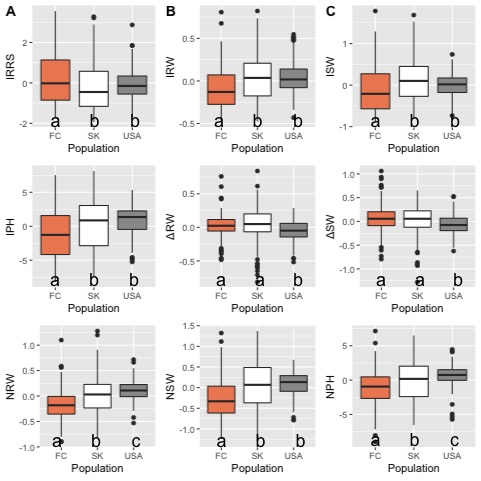

Supplement: S8 Fig — The GRIN population (red) the SK population (white) boxplots, and the US population (grey) populations are represented for all nine traits: IRRS, inoculated root rot score; IRW, inoculated root weight; ISW, inoculated shoot weight; IPH, inoculated plant height; ΔRW, change in root weight; ΔSW, change in shoot weight; NRW, non-inoculated root weight; NSW, non-inoculated shoot weight; NPH, non-inoculated plant height. Significantly differences in population were tested using Fisher’s protected LSDs (P value <0.05) are indicated with letters. (TIFF) [file pone.0227710.s016.tiff]

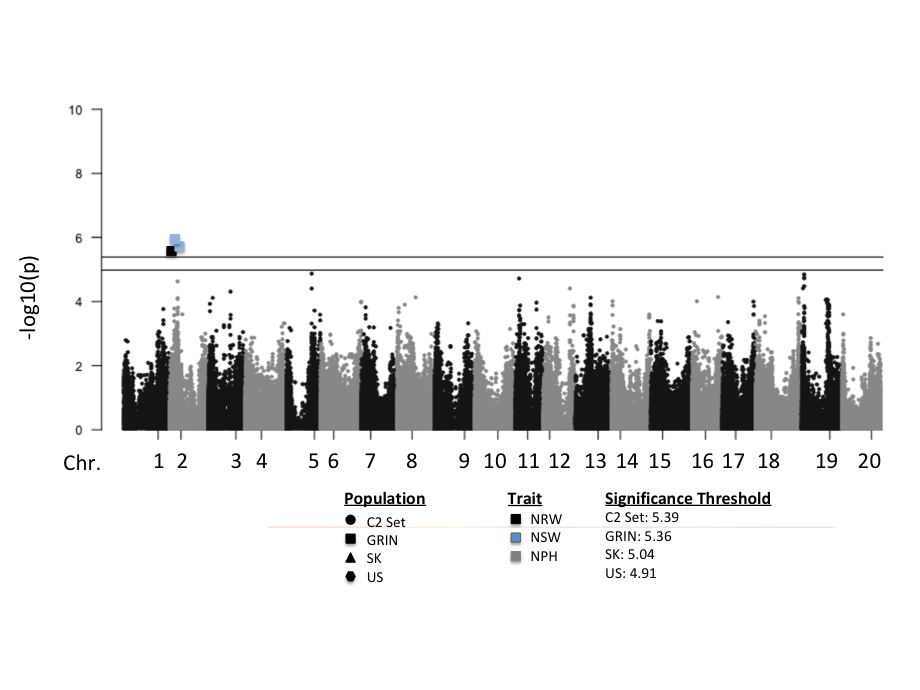

Supplement: S9 Fig — Population the marker-trait association was identified in via the shape of the significant marker. The specific trait is identified by the color of the marker. Significance thresholds calculated using SimpleM are displayed for the C2 set, and the C2-US Population. (TIFF) [file pone.0227710.s017.tiff]

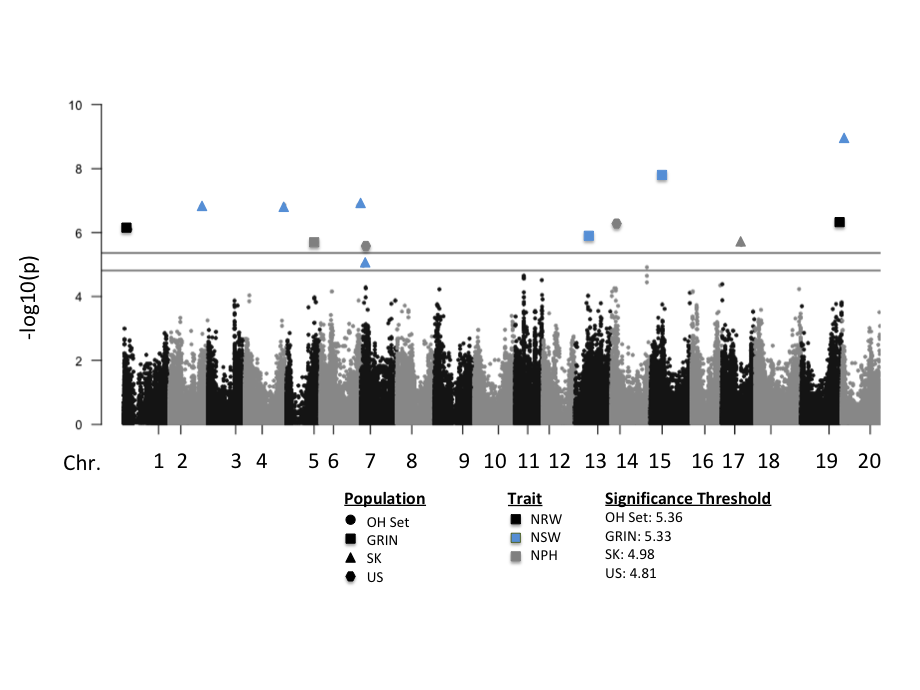

Supplement: S10 Fig — The figure shows which population the marker-trait association was identified in via the shape of the significant marker. The specific trait is identified by the color of the marker. Significance thresholds calculated using SimpleM are displayed for the OH set, and the OH-US Population. (TIFF) [file pone.0227710.s018.tiff]
